# A Novel Interception Strategy in a Miniature Robber Fly with Extreme Visual Acuity

**DOI:** 10.1016/j.cub.2017.01.050

**Published:** 2017-03-20

**Authors:** Trevor J. Wardill, Samuel T. Fabian, Ann C. Pettigrew, Doekele G. Stavenga, Karin Nordström, Paloma T. Gonzalez-Bellido

**Affiliations:** 1 Department of Physiology, Development and Neuroscience, University of Cambridge, Downing Street, Cambridge CB3 2EG, UK; 2 Eugene Bell Center for Regenerative Biology and Tissue Engineering, MBL, 7 MBL Street, Woods Hole, MA 02543, USA; 3 Leader Heights Animal Hospital, 199 Leaders Heights Road, York, PA 17402, USA; 4 Computational Physics, Zernike Institute for Advanced Materials, University of Groningen, Groningen 9747 AG, the Netherlands; 5 Centre for Neuroscience, Flinders University, GPO Box 2100, Adelaide, SA 5001, Australia

**Keywords:** vision, invertebrate, retina, spatial resolution, predation, flight, interception strategy, moving target, tracking

## Abstract

Our visual system allows us to rapidly identify and intercept a moving object. When this object is far away, we base the trajectory on the target’s location relative to an external frame of reference [1]. This process forms the basis for the constant bearing angle (CBA) model, a reactive strategy that ensures interception since the bearing angle, formed between the line joining pursuer and target (called the range vector) and an external reference line, is held constant [2, 3, 4]. The CBA model may be a fundamental and widespread strategy, as it is also known to explain the interception trajectories of bats and fish [5, 6]. Here, we show that the aerial attack of the tiny robber fly *Holcocephala fusca* is consistent with the CBA model. In addition, *Holcocephala fusca* displays a novel proactive strategy, termed “lock-on” phase, embedded with the later part of the flight. We found the object detection threshold for this species to be 0.13°, enabled by an extremely specialized, forward pointing fovea (∼5 ommatidia wide, interommatidial angle Δφ = 0.28°, photoreceptor acceptance angle Δρ = 0.27°). This study furthers our understanding of the accurate performance that a miniature brain can achieve in highly demanding sensorimotor tasks and suggests the presence of equivalent mechanisms for target interception across a wide range of taxa.

**Video Abstract:**

## Highlights

• *Holcocephala fusca* robber flies capture prey with a constant bearing angle strategy
• The approach is proactively altered to “lock on” on to prey within 29 cm distance
• The retina spatial resolution is 0.28°, but the object detection threshold is 0.13°
• The fly’s stereopsis range is estimated to be ∼26 cm

## Graphical Abstract

**Figure:**
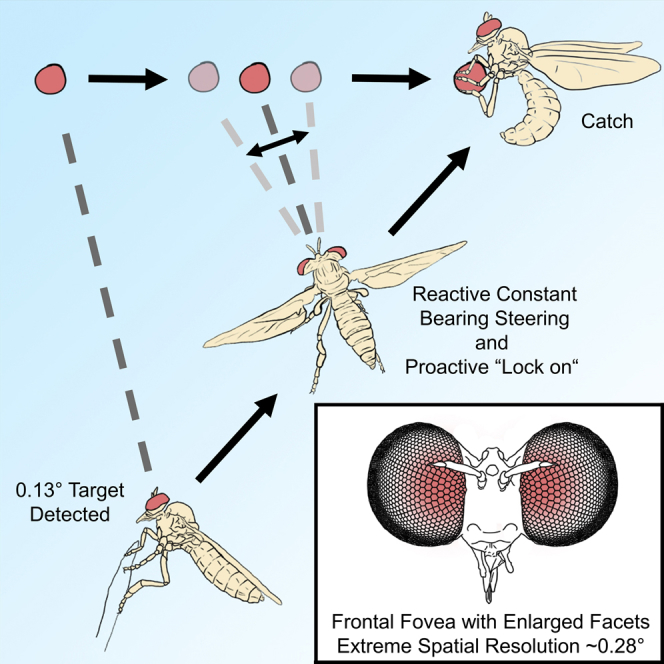

## Teaser

Robber flies are aerial predators. Here, Wardill, Fabian, et al. show that the tiny robber fly *Holcocephala fusca* attacks a detected prey reactively but proactively changes its speed and direction when the prey is within 29 cm. They also show that the very small object detection threshold of 0.13° is supported by a striking visual fovea.

## Results

### Aerial Attack Strategy

**Figure 1:**
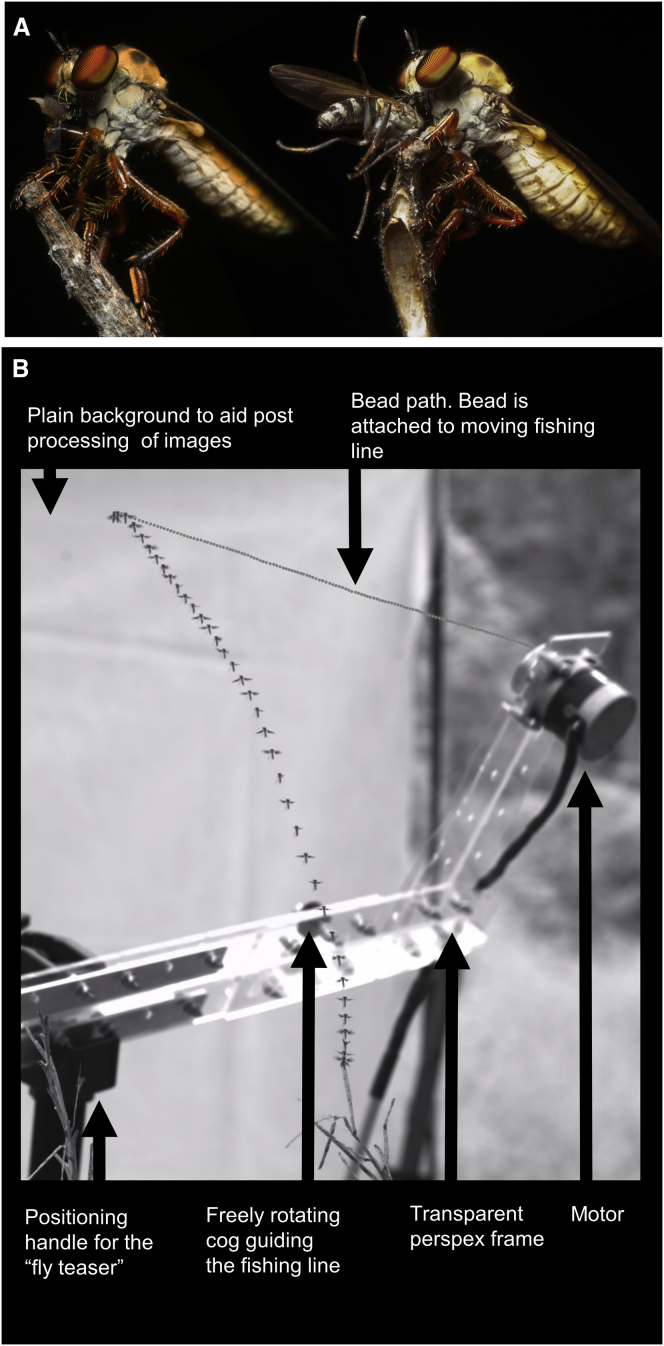
*Holcocephala* with Prey Items and Test of *Holcocephala*’s Predatory Behavior (A) Two examples of *Holcocephala* feeding on prey caught mid-air with the smallest and largest prey that we observed. (B) The “fly teaser” ensemble, used to entice flies to attack artificial targets in their natural environment, which allowed for controlled stimulus parameters, such as size, distance, and speed. Overlay: simultaneous positions of the fly and bead throughout the trajectory.

**Figure 2:**
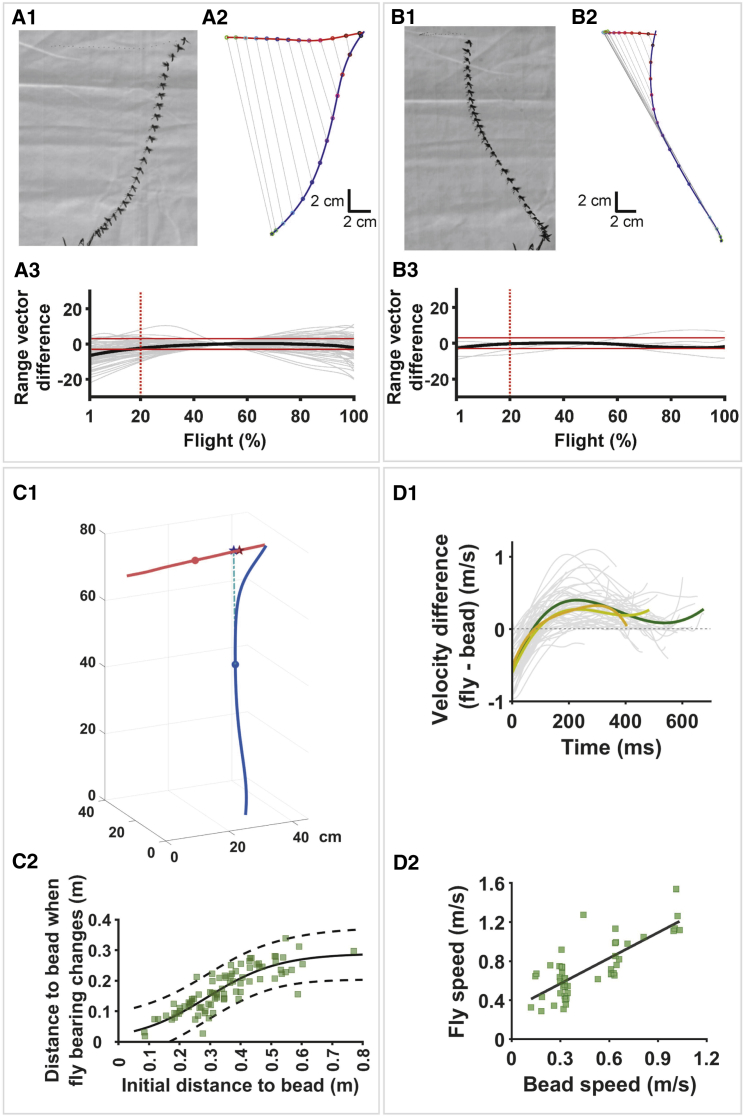
Geometry and Timing of the *Holcocephala* Aerial Attack (A1) *Holcocephala* flight trajectory toward a target moving at constant speed. (A2) 3D reconstructed trajectory of the flight course (blue curve) showing nearly parallel range vectors of decreasing length (target trajectory: red curve). (A3) The difference in direction (in degrees) between any one range vector (the line joining predator and prey at each frame) and the median range vector for the trajectory plotted for all trials in which *Holcocephala* chased a target moving at constant speed (n = 63; solid red lines = −3° and +3°; dotted red line: 20% of flight time elapsed; see also Figure S1). (B1 and B2) Flight trajectory when the presented bead changes velocity and completely reverses direction, during which *Holcocephala* maneuvers to keep the range vector parallel (see also Movie S1). (B3) During bead reversal presentations, the difference between the range vectors and the median vector stays close to zero (n = 4). (C1) Trajectory that would have resulted in a head-on collision interception (cyan dashed line), but before the collision *Holcocephala* arched backward (blue line). (C2) Distance to target when the change in heading occurs (black line: four-parameter sigmoidal fit; adjusted r^2^ = 0.73; 95% confidence bounds shown by broken lines; n = 86). (D1) The difference in velocity between fly and bead. After the initial phase, the flies stop accelerating and keep their speed at a value that is slightly higher than that of the bead; this behavior is independent of attack duration (average short, medium, and long trajectories shown in short orange, medium lime, and long green lines, respectively). (D2) Fly speed as a function of bead speed. The average velocity during the lock-on phase is correlated with that of the bead (adjusted r^2^ = 0.6; for all D plots, n = 51). See also Figure S4 and Movie S2.

In our study of the aerial hunts of the robber fly *Holcocephala* (Figure 1), we considered whether its behavior is consistent with the constant bearing angle (CBA) model (Figure S1). We tested this on flies in their natural habitat by presenting a range of beads (diameter 1.3, 2.9, and 3.9 mm) on a fishing line, whose speed was controlled by a stepper motor (Figure 1B; Supplemental Experimental Procedures). We recorded the flies’ behavior with two high-speed video cameras and reconstructed their flight trajectory in three dimensions. Consistent with the CBA model, we found that when pursuing a bead moving at constant speed, the range vectors were close to being parallel across most of the trajectory: for 80% of the flight time, the absolute difference between each range vector and the trajectory median range vector was on average less than 3° (Figures 2A1–2A3; n = 63 attacks to a 1.3 mm bead). By applying proportional navigation, the guidance law associated with the CBA model [4, 7], a pursuer can control the necessary steering command to null any change in the velocity of the target, thereby keeping the range vectors parallel and actively maintaining the CBA (Figures S1G and S1H). We tested the CBA mechanism of *Holcocephala* by decelerating or reversing the bead during the attack (Movie S1). We found that *Holcocephala* compensates for bead trajectory changes and actively keeps the range vectors parallel (Figures 2B1–2B3; n = 4 attacks to a 1.3 mm bead), consistent with achieving a CBA through proportional navigation.

One surprising finding was that the latter part of *Holcocephala*’s pursuing trajectory was distinctly curved. This was most apparent when the targeted bead traveled toward the front of the animal and *Holcocephala* took off with a “head-on” collision course but ultimately intercepted the bead while flying backward (Figure 2C1; seen in 22 of the 63 analyzed trajectories toward the 1.3 mm bead). Under a CBA strategy, compensatory flight alterations are necessary if the prey alters its velocity (direction, speed, or both), but since the targets were presented with constant velocity, the fly’s change in direction was not elicited by the target. Could *Holcocephala* simply have miscalculated the heading necessary for a straight interception or perhaps failed to attain the speed necessary to intercept with such a heading? This is unlikely, as extending the initial *Holcocephala* trajectory along the velocity vector attained just before the change in heading (Figure 2C1, turquoise broken line) shows that *Holcocephala* would have been very near the interception point (minimal distance 2.9 ± 0.4 cm, mean ± SE, n = 22). Presumably this small error could have been easily corrected during the rest of the attack. Nonetheless, the observed curved trajectory added to the total interception time on average 132 ± 7 ms (mean ± SE) until contact, which is a substantial 27% ± 1% of the total flight time (mean ± SE, n = 22).

To understand what induced the change in heading, we first looked at its timing. Our analysis showed that the change in heading was accompanied by a deceleration. We therefore took the first time point in the trajectory where a deceleration was detected as an objective measure for the start of the change in heading. By fitting the data with a sigmoidal curve (trajectories to beads of all three sizes in which a clear deceleration was detected were included; n = 86), we found that independent of the starting distance, the maximum distance between target and fly at which the change occurred was 29 ± 4 cm (95% confidence bounds; R^2^ adjusted = 0.73; Figure 2C2). We also found that shortly after the change in heading, *Holcocephala* fixed its forward velocity vector slightly above that of the bead (Figure 2D). Our results reflect a “lock-on” process, initiated by information that becomes available once the fly is within ∼29 cm of the target. Here, we use “lock-on” to refer to the phase during which the fly has a new heading and the speed is fixed to a value slightly higher than that of the prey. The different mechanisms that could underlie this behavior are addressed in the discussion.

### Minimum Behavioral Discrimination of a Moving Target—Single Object Threshold—and Acuity Parameters of *Holcocephala*’s Fovea

**Figure 3:**
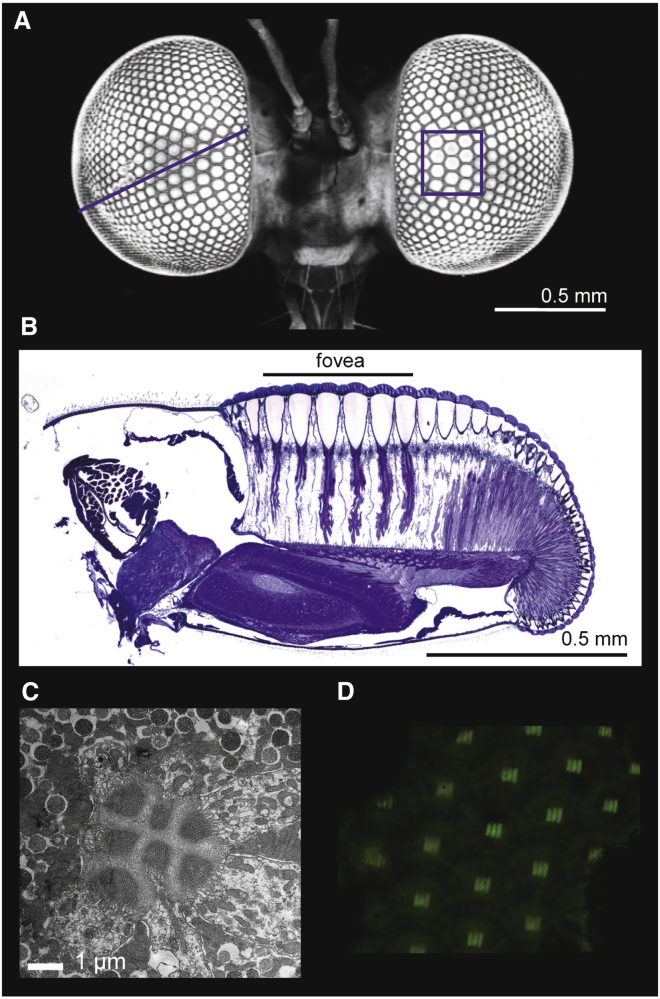
Structural Specializations of the *Holcocephala* Eye (A) Two-photon image of the *Holcocephala* head showing the enlarged frontal facets. The line and box mark the locations of the oblique section shown in (B) and the cross-section shown in (C), respectively. (B) Oblique eye section showing the acute zone with enlarged sizes and focal lengths of the facet lenses, as well as a flat cornea and basement membrane. (C) Cross-section of an ommatidium in the acute zone showing the rhabdomeres with tip diameter ∼0.9 μm. (D) Image of a grating pattern created by an isolated cornea with the hanging drop method, which allowed calculation of the focal length (*f*).

The five longest target detection distances in our CBA experiments that lead to a successful catch of the 1.3 mm bead were > 53 cm (for example, Movie S2), and at such distances the bead subtended no more than 0.12°–0.14° on the retina. Therefore, the minimum single object threshold resolved by *Holcocephala*’s visual system must be as small as ∼0.13°. The *Holcocephala* eye has an ommatidial lattice with an excessive gradient in facet size, which indicates the presence of a fovea with an extreme degree of functional regionalization. We therefore investigated the internal anatomy, to further elucidate the retinal adaptations that provide the necessary spatial visual performance driving the fly’s pursuit behavior. Sectioning the *Holcocephala* eye revealed a reduced curvature of the frontal cornea as well as flattening of the basement membrane. The extremely enlarged frontal ommatidia have facet lenses with extended focal lengths that focus incident light into unusually slender rhabdomeres (Figure 3). These specializations are known to optimize the spatial resolution of fly eyes, thus creating an area of high acuity, a fovea [8].

Crucial measures for the spatial acuity of a compound eye are the photoreceptor acceptance angle, Δρ, and the interommatidial angle, Δφ, the angle between the optical axes of neighboring ommatidia. The photoreceptor acceptance angle can be estimated from the ratio of the rhabdomere diameter (*D*_r_) and the facet lens’s focal length (*f*). Anatomical measurements yielded *D*_r_ = 0.92 ± 0.13 μm (Figure 3C). Using the hanging drop method with cleaned corneas in the eye region with the largest facet lenses (Figure 3D), the focal length was found to be *f* = 190 ± 4 μm, hence yielding a very small photoreceptor acceptance angle: Δρ = 0.28 ± 0.04°. Measurement of the interommatidial angle with the preferred pseudopupil method [9] was problematic due to *Holcocephala*’s dense eye pigmentation, and therefore we considered the unique property of fly eyes where sets of six photoreceptors located in six adjacent ommatidia pool their signals in one cartridge of the lamina, the first optical ganglion below the retina. This neural superposition principle dictates that the interphotoreceptor angle, the angle between the visual axes of the photoreceptors within one and the same ommatidium, equals the local interommatidial angle. Yet, in a detailed study on a number of fly species, Pick [10] demonstrated that the interphotoreceptor angle is actually ∼20% larger than the interommatidial angle. The interphotoreceptor angle in an ommatidium equals the ratio of the distance between adjacent rhabdomeres and the focal length of the facet lens. From ultrathin cross-sections of the eye region with the largest facet lenses (see Supplemental Experimental Procedures), we found that the interrhabdomere distance was *D*_i_ = 1.15 ± 0.16 μm, thus yielding the interphotoreceptor angle *D*_i_ /*f* = 0.35° ± 0.05°, or, applying Pick’s [10] correction, the interommatidial angle becomes Δφ = 0.28° ± 0.04°.

### Arrangement of Interommatidial Angles and Direction of Visual Axes in the Fovea

**Figure 4:**
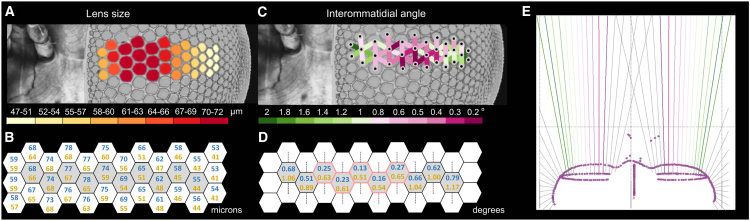
Optical Characteristics of the *Holcocephala* Fovea (A) Distribution of the average diameters of the foveal facet lenses (values in look-up table in μm; mean of n = 4). (B) Lens diameter (in μm) for a large fly (blue) and a small fly (yellow). (C) Interommatidial angles derived from two-photon microscopy images (in degrees; n = 4); see also Figures S2 and S3. (D) Range of vertical interommatidial angles (in degrees; n = 4); blue and yellow values are mean ± 0.19°. (E) Ommatidial axes (colored lines) from a *Holcocephala* sample. The dark pink lines denote the centers of the acute zones where the axes of the central ommatidia are virtually parallel; the rest of the ommatidial axes diverge progressively. The dotted gray lines, indicating the additional ommatidial axes outside the fovea, were not measured but are added to heuristically illustrate the whole visual field. See also Movie S3.

To understand the distribution of the visual axes across the fovea, we acquired two-photon microscopy images of the fovea region, yielding 3D anatomical stacks of the eye fovea’s anatomy (Movie S3). The fovea appeared to be ∼5 ommatidia wide, with diameter of the facet lenses 70–78 μm (Figures 4A and 4B) and interommatidial angles Δφ = 0.40° ± 0.19° (Figures 4C and 4D; Figures S2 and S3; Supplemental Experimental Procedures). The interommatidial angles deduced above (Δφ = 0.28° ± 0.04°) are within this range. The two-photon microscopy images further demonstrated that the central ommatidia of the acute zones in the two eyes have virtually parallel visual axes, meaning that *Holcocephala* has a binocular view of the world (Figure 4E). In summary, the behavioral performance that we measured (>53 cm interception distances and ∼0.13° object threshold) is supported by the fine spatial resolution (0.28°) provided by the specialized fovea.

## Discussion

We have investigated the predatory attack of the robber fly *Holcocephala fusca* and found that this species generates an interception course using a constant bearing angle strategy and applying maximum acceleration to quickly get closer to the prey. It is not surprising that *Holcocephala* utilizes a CBA strategy during most of its flight, as this reactive strategy enables compensation for (1) unexpected changes in the target’s velocity and (2) uncertainties about the perceived location, size, and speed of the target when absolute depth cues are absent (Figure S4). Even humans, who arguably have higher computational brain power, rely on a CBA to solve similar tasks [1]. This indicates that the CBA strategy is a robust way to intercept targets when sensory information is limited, independent of the processing power available. To further confirm the use of proportional navigation, future studies will need to embed this guidance law in a control architecture that can match entire trajectories. It is also of importance to note that the use of a CBA reactive strategy does not exclude other guiding principles from being applied. For example, during their interception flights, dragonflies use both reactive and proactive motor commands [11]. It is therefore of interest that once *Holcocephala* is within ∼29 cm of the target, it implements a heading and speed change. The presence of such a lock-on phase has not yet been described in any other flying animal. Although the lock-on strategy extends the total flight time (Figure 2), lowering the final flight speed needed for interception and extending the time over which *Holcocephala* may catch the prey is likely a highly effective adaptive behavior, consistent with the priority to ensure highest success rates in the face of sensorimotor delays and errors. The resulting strategy is similar to that of a baton pass in a relay race: a pass between two runners with similar direction and velocity is more likely to be successful than one between two runners passing each other in opposite directions.

What mechanisms could explain the trigger of the lock-on phase? It is possible that the lock-on phase is driven by invariant properties of the image, and not by actual distance estimation. For example, the escape responses of locusts, frogs, fruit flies, and crabs to a looming stimulus occur after the target reaches a certain angular size threshold [12, 13, 14, 15]. However, when testing the lock-on phase by offering beads with different sizes, we found that at the moment the lock-on phase was initiated, the angular size of the target varied significantly (large bead: 1.23° ± 0.66°, medium bead: 0.81° ± 0.22°, small bead: 0.52° ± 0.67°, mean ± SD; n = 14, 9, and 63, respectively; p = 0.001 ANOVA). Therefore, the trigger for the lock-on phase is unlikely to be a specific subtended angular size. Nevertheless, similar to flies initiating deceleration prior to landing [16], *Holcocephala* may have used as the trigger the angular size of the object over its rate of expansion. This ratio, often referred to as optical tau [17], provides an estimate for time to contact and can be used as a threshold for initiation of a motor command with appropriate timing [18]. However, the *Holcocephala* attack violates two conditions that must be met for optical tau to be reliable: constant approach speed and symmetrical head-on approach [16]. Moreover, optical tau obtained from a target subtending a small size is unreliable [19] because the calculation depends on the perceived expansion rate. At the maximum distance at which the lock-on phase is initiated (∼29 cm), the 1.3 mm bead subtends ∼0.26°. With a foveal interommatidial angle of 0.28°, the bead will be detected by at most two ommatidia. Although it is conceivable that the rate of change in light intensity falling on a single ommatidium may act as an “expansion” parameter, to our knowledge, animals performing aerial pursuits do not exploit the contrast change in a single light detector as a reliable cue to calculate time to contact. Likewise, by translating or pivoting, an array of parallel sensors can provide depth and range information [20], and a similar mechanism cannot be excluded without further analysis.

Alternatively, distance estimation via stereopsis could underlie the trigger of the lock-on phase in *Holcocephala*. Stereopsis is the reconstruction of depth from the disparity in the two ocular images due to the distance between the eyes [21]. If the visual fields of both eyes overlap sufficiently, the stereopsis range is solely dependent on the resolution of the retina and the distance between the forward-facing foveas [22]. Indeed, short range stereopsis has been demonstrated in mantids [23, 24, 25], and extended stereopsis has been predicted in mantid and dragonfly larvae with stereopsis ranges 46 and 26 cm, respectively [26]. *Holcocephala* also has a binocular field of view (Figure 4E). Given that the largest *Holcocephala* sample in our collection has an inter-fovea distance of 1.3 mm, with an interommatidial angle Δφ = 0.28° the limit of the stereopsis range is 26 cm. Given the small photoreceptor angle of Δρ = 0.27°, it may be feasible that *Holcocephala* uses depth cues provided by stereopsis to trigger the lock-on phase. Stereopsis at such range would require that the target be foveated, and it is possible that the head movements exhibited by *Holcocephala* prior to launching an attack serve such purpose. Whether or not *Holcocephala* uses time-to-contact acquired from monocular or binocular cues, the process bears parallels to the strategies employed by humans. For instance, when carrying out a long range interception, humans use both optical tau [27] and binocular cues [28] to improve the performance of reaching and grasping movements, and such prehensile movements form the second phase of a given task.

The existence of a localized area of high resolution in compound eyes, also called acute zone, dorsal zone, or love spot, is well documented among insect species that depend on target tracking for survival or mating [29], but the *Holcocephala* fovea clearly provides an extreme case. For example, the ∼20 foveal ommatidia occupy ∼20% of the eye volume (Figure 3) and span ∼0.1% of the eye’s visual space (angular range < 4.5°; Figure 4). In summary, our behavioral results of *Holcocephala* and the anatomical and optical data of its eyes demonstrate the extremely specialized visual capacities of a very small robber fly. Our findings may provide the basis of bioinspired guidance systems in miniature, aerial and autonomous vehicles, where maximum performance with minimum size is highly desirable.

## Author Contributions

A.C.P. searched for and located the animals and reported their biology ahead of the experimental tests. T.J.W. developed the methodology for the behavioral experiments and two-photon imaging. T.J.W. and P.T.G.-B. designed the behavioral experiments, and T.J.W., S.T.F., and P.T.G.-B. conducted them. S.T.F. analyzed all other behavioral data. T.J.W., P.T.G.-B., and S.T.F. acquired the microscopy data. S.T.F. and P.T.G.-B. analyzed it. D.G.S. provided assistance in the optical calculations. K.N. collaborated in analyzing the results. All authors contributed to critical discussions and the writing of the manuscript.

## Supplemental Information

Document S1. Supplemental Experimental Procedures and Figures S1–S4

Movie S1. Bead Reversal to Test Flight Control, Related to Figure 2 *Holcocephala* takes off when the bead is moving forward. Shortly after, the bead stops and reverses in direction. During flight, *Holcocephala* compensates reactively for such changes, which leads to a successful target interception. Each frame equals one millisecond.

Movie S2. Testing the Object Detection Threshold of *Holcocephala*, Related to Figure 2 This video shows the second longest distance (59 cm) at which *Holcocephala* detected and subsequently successfully intercepted the 1.3 mm bead. Each frame equals one millisecond.

Movie S3. 3D Image of a *Holcocephala* Head, Related to Figure 4 The cuticle’s autofluorescence signal is particularly strong, and the ommatidial lattice can be seen with ease. The autofluorescence signal arising from the photoreceptors is weaker, but it is sufficient to locate the photoreceptor tips. The sample was bleached and cleared before being imaged with a two-photon microscope.

Document S2. Article plus Supplemental Information
