# Supplementary figures and images for: A Novel Interception Strategy in a Miniature Robber Fly with Extreme Visual Acuity
Source: Curr Biol. 2017 Mar 20;27(6):854–9. doi: 10.1016/j.cub.2017.01.050 (PMC5364399; doi:10.1016/j.cub.2017.01.050)

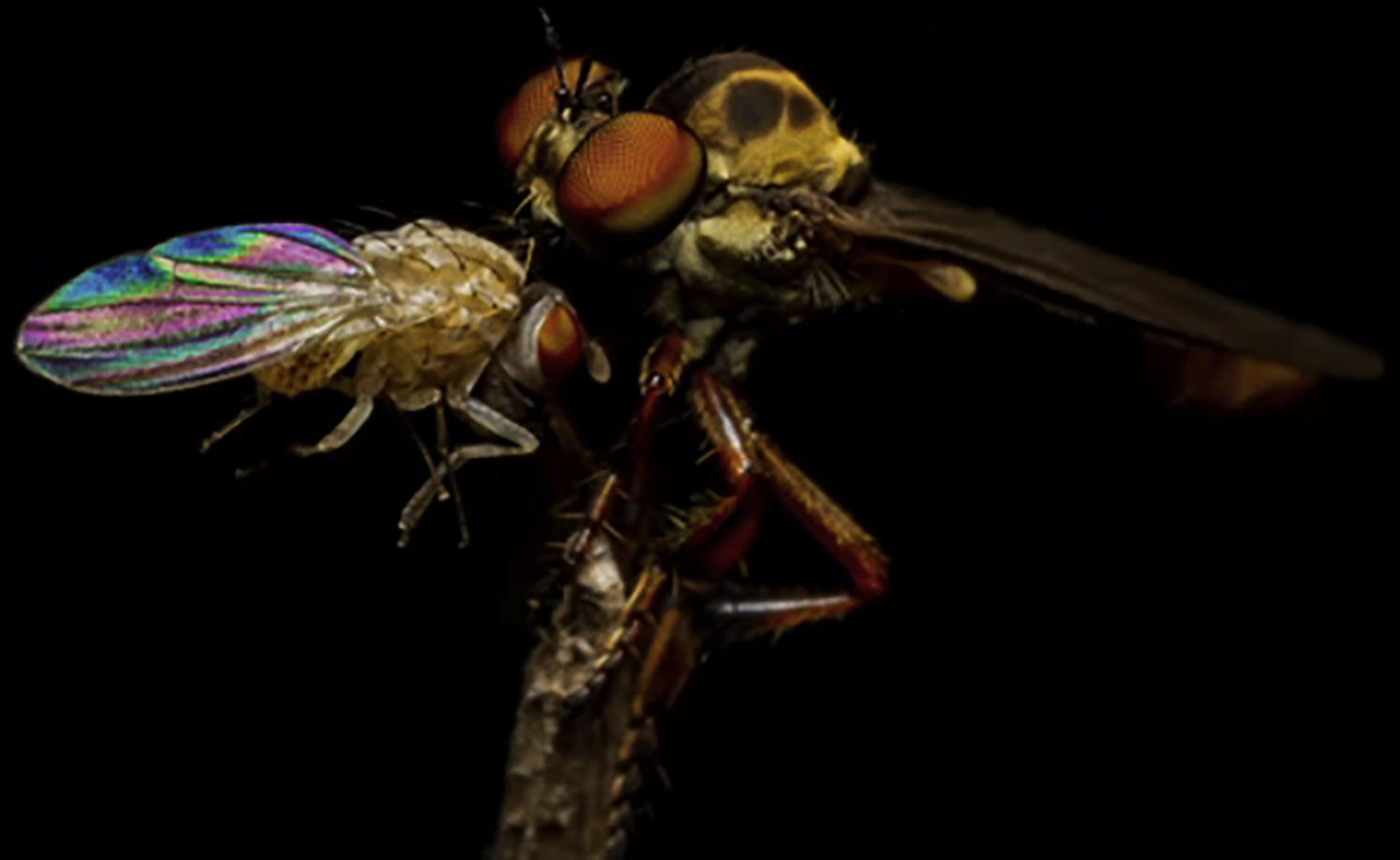

Supplement: Supplementary file 1 [file mmc6.jpg]

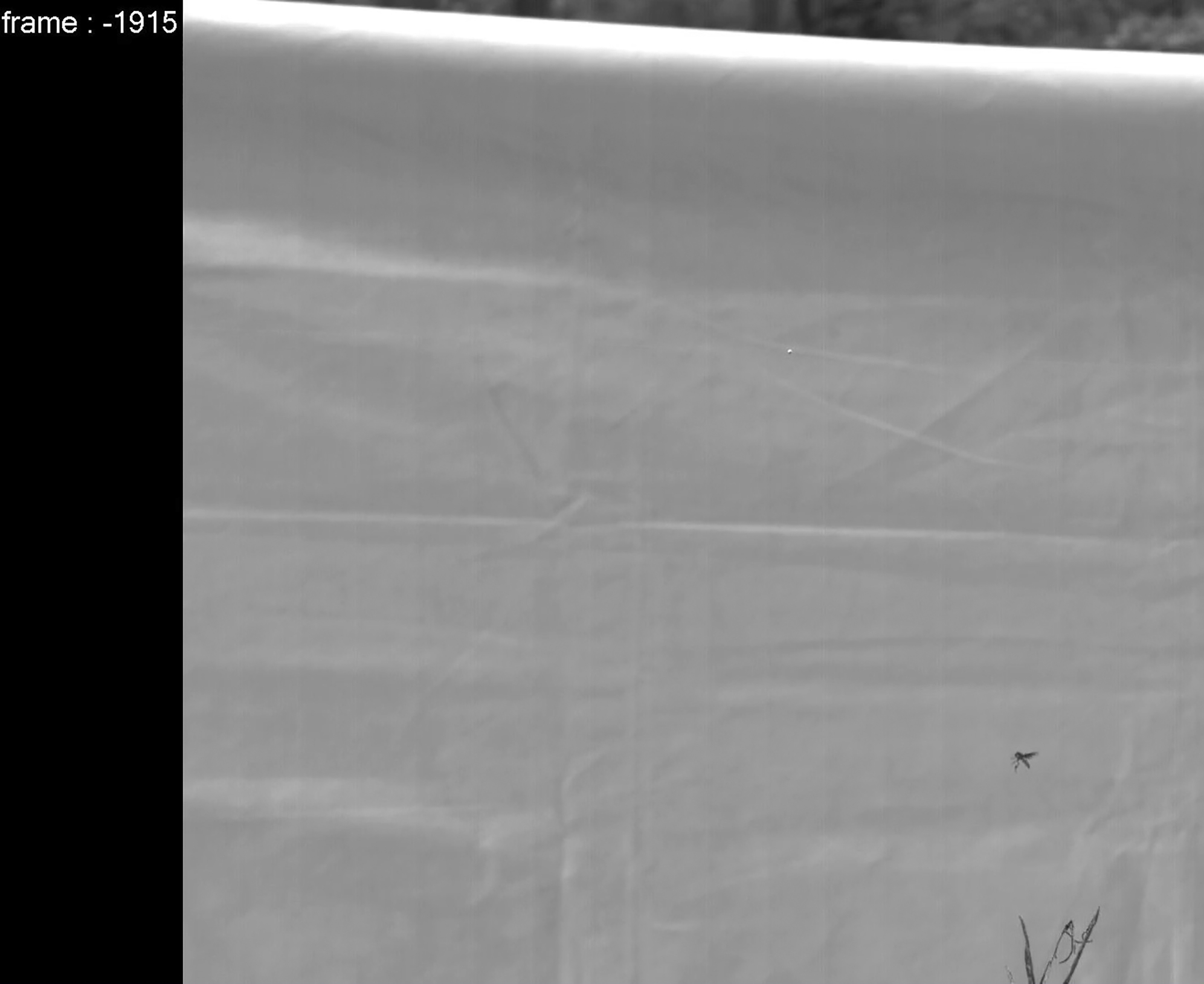

Supplement: Movie S1. Bead Reversal to Test Flight Control, Related to Figure 2 — Holcocephala takes off when the bead is moving forward. Shortly after, the bead stops and reverses in direction. During flight, Holcocephala compensates reactively for such changes, which leads to a successful target interception. Each frame equals one millisecond. [file mmc2.jpg]

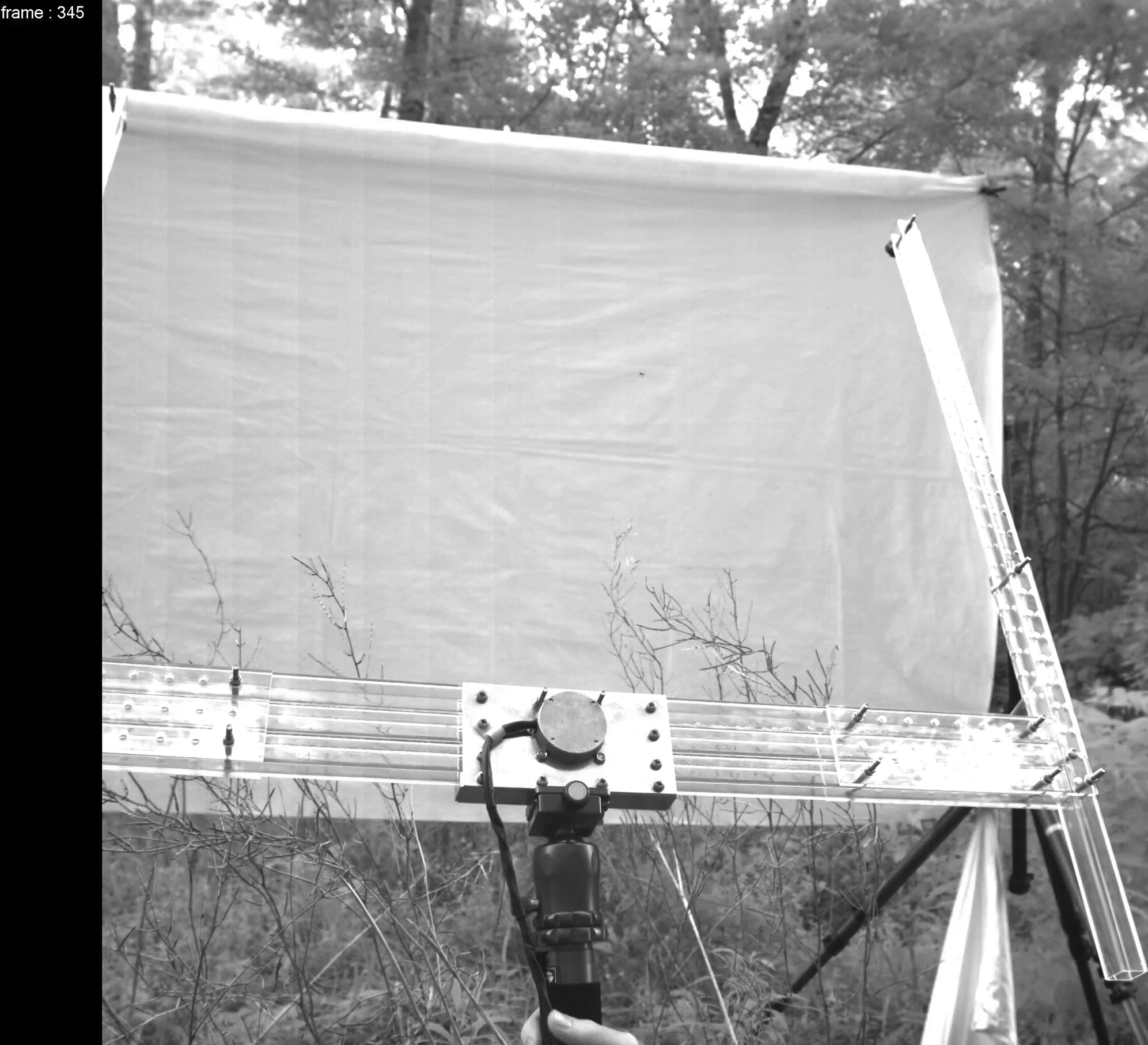

Supplement: Movie S2. Testing the Object Detection Threshold of Holcocephala, Related to Figure 2 — This video shows the second longest distance (59 cm) at which Holcocephala detected and subsequently successfully intercepted the 1.3 mm bead. Each frame equals one millisecond. [file mmc3.jpg]

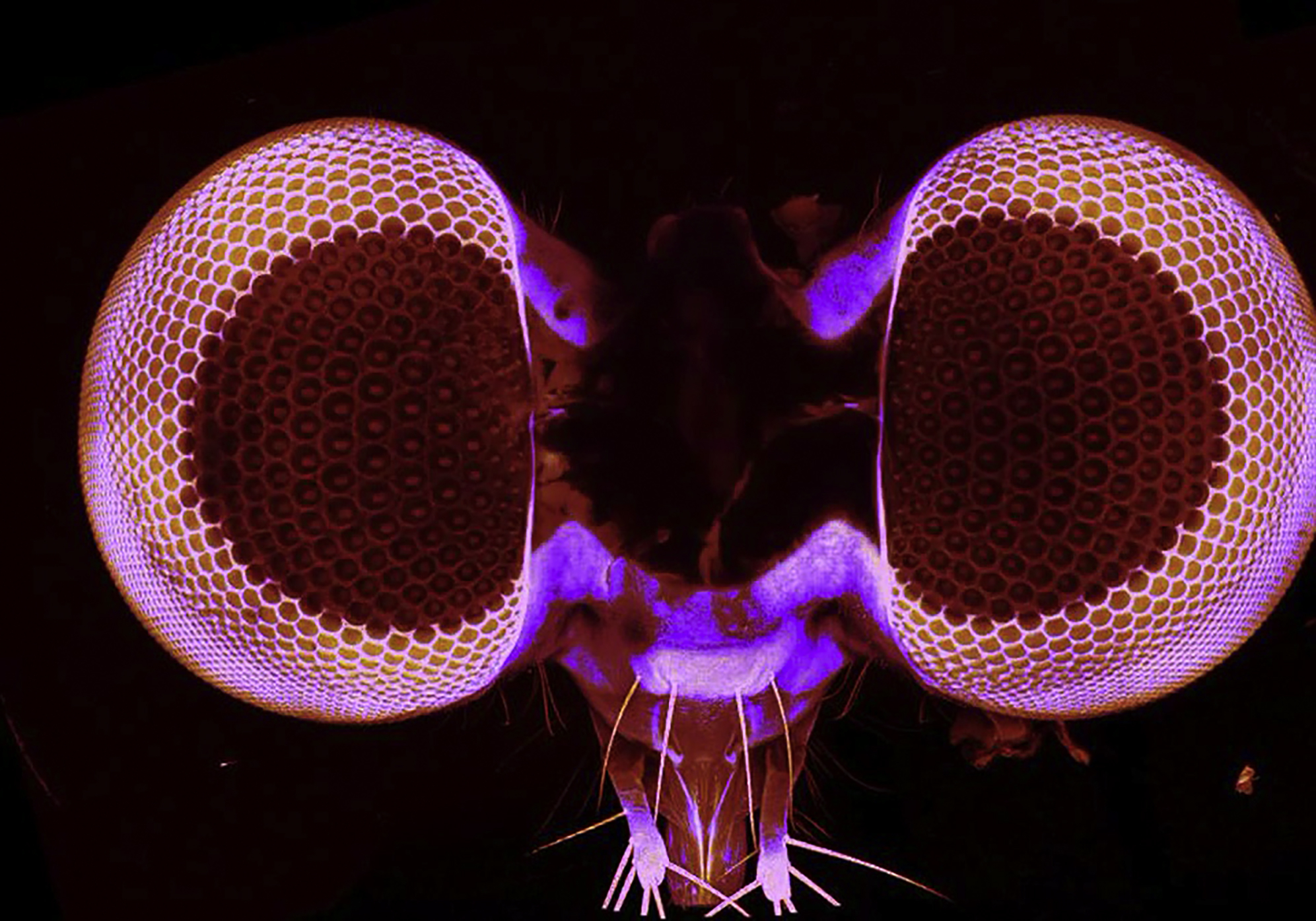

Supplement: Movie S3. 3D Image of a Holcocephala Head, Related to Figure 4 — The cuticle’s autofluorescence signal is particularly strong, and the ommatidial lattice can be seen with ease. The autofluorescence signal arising from the photoreceptors is weaker, but it is sufficient to locate the photoreceptor tips. The sample was bleached and cleared before being imaged with a two-photon microscope. [file mmc4.jpg]
