# Supplementary material for: A Novel Interception Strategy in a Miniature Robber Fly with Extreme Visual Acuity
Source: Curr Biol. 2017 Mar 20;27(6):854–9. doi: 10.1016/j.cub.2017.01.050 (PMC5364399; doi:10.1016/j.cub.2017.01.050)
Supplement: Document S1. Supplemental Experimental Procedures and Figures S1–S4 [file mmc1.pdf]

Current Biology, Volume *27*

Supplemental Information

A Novel Interception Strategy in a Miniature Robber Fly with Extreme Visual Acuity

Trevor J. Wardill, Samuel T. Fabian, Ann C. Pettigrew, Doekele G. Stavenga, Karin Nordström, and Paloma T. Gonzalez-Bellido

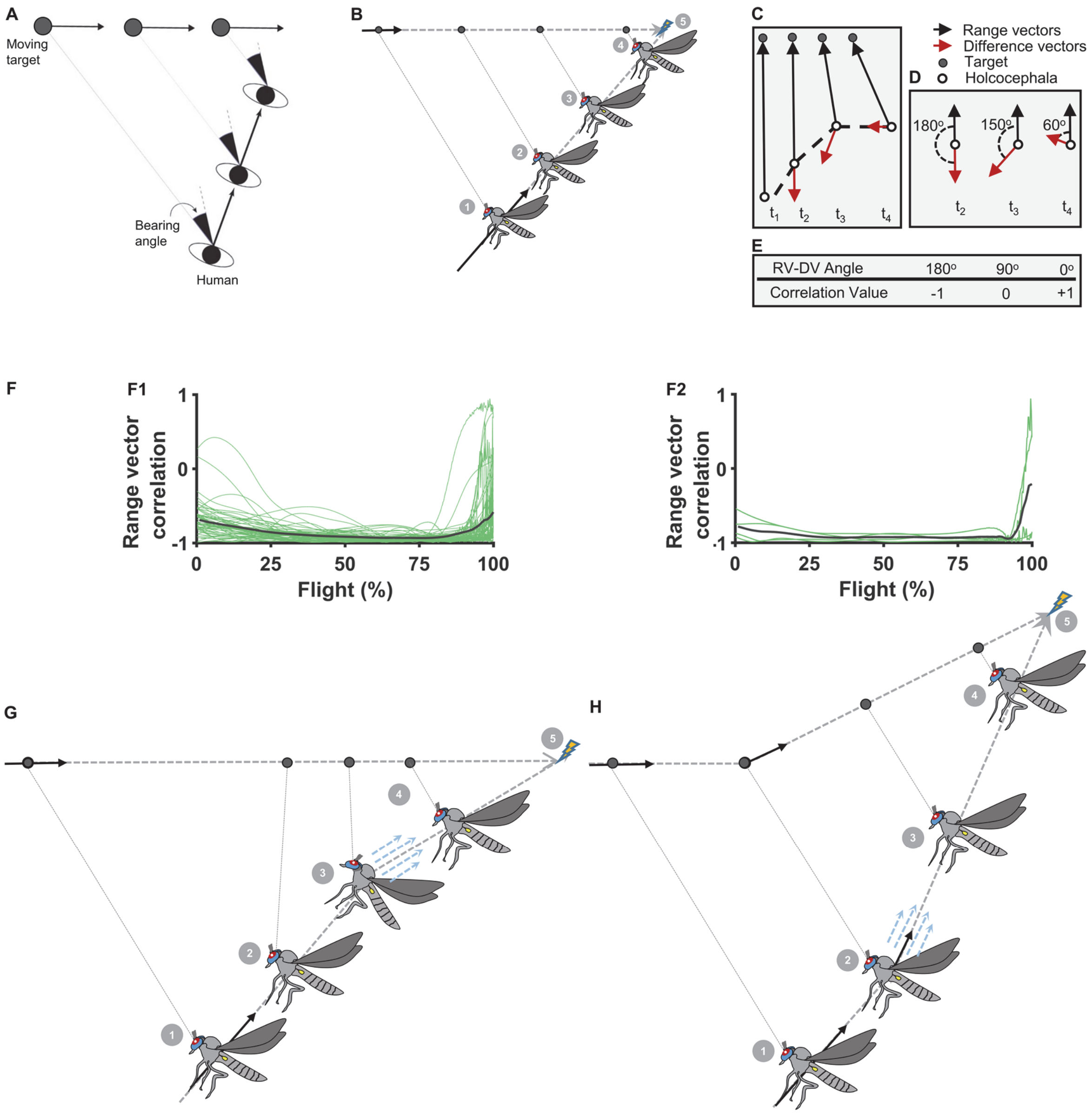

A
Moving target
Bearing angle
Human
B
C
Range vectors
Difference vectors
Target
Holcocephala
D
180º
150º
60º
E
RV-DV Angle
180º
90º
0º
Correlation Value
-1
0
+1
F
F1
F2
Range vector correlation
Flight (%)
G
H

Figure S1. Related to Figure 2. Diagrams showing how the constant bearing angle strategy (CBA) and proportional navigation can be used to intercept targets. (**A**) Top view diagram of human (head shown in black, shoulders in white) intercepting a ball moving on the ground. The constant bearing angle (CBA) strategy results in parallel range vectors (grey line between human and ball, also called the line of sight) that decrease in range. Reproduced from [S1], with permission from the author. (**B**) *Holcocephala* can also intercept a moving target by employing the constant bearing angle strategy: (1) When the prey flies with constant velocity and direction *Holcocephala* flies backwards to intercept it. (2) *Holcocephala* maintains its own heading and speed by maintaining the image of the prey on the same retina location. (3) This approach allows *Holcocephala* to maintain an interception heading, without explicitly knowing where the interception will take place. (4) At close range, the prey does not detect the *Holcocephala*, or does not have time to react. (5) The target is caught in a few more milliseconds. (**C**) To allow for comparison across studies, we also calculated the vector correlation as done by [S2]. For every frame in this trajectory, the difference vector is calculated (red). The difference vector is the difference between any one range vector and a previous range vector. (**D**) To calculate correlation between vectors, at every frame the range vector is subtracted form the difference vector (angle shown = RV - DV angle). As can be seen, when simply closing range and not changing angle, the difference vector is at 180° to the range vector. However, the difference vector becomes less than 180° when there are discrepancies in direction, as in t_3. When range is gained between fly and bead, the angle between the difference vector and the range vector falls below 90°. (**E**) The difference between the range vector (RV) and difference vector (DV) is then normalized (-1 to +1). This correlation value gives a proxy of how parallel the range vectors are between each other. (**F**) The resulting vector correlation value of trials in which the bead moved with a constant speed (F1) and those in which it decelerated or even reversed (F2). (**G**) Following alterations in the prey's bearing or velocity, proportional navigation can be used by *Holcocephala* to constantly update its own bearing and achieve interception without explicit knowledge of the time or point at which contact will occur: (1) The prey flies with constant velocity and direction. *Holcocephala* flies backwards to intercept it. (2) The target accelerates, and its image slips out of the acute zone. (3) *Holcocephala* compensates immediately by changing the tilt of its head, or the tilt of its head and body together. This action brings the image of the prey back to the acute zone. Simultaneously, the fly applies a proportional change to the rotation rate of its velocity vector, which results in a new heading direction. (4) *Holcocephala* stabilizes the range vector once more, and body position and the head are brought back to their original orientation. (5) The target is caught in a few more milliseconds. (**G**) The same process applies when a target changes direction: (1) The prey flies with constant velocity and direction. *Holcocephala* flies backwards to intercept it. (2) The prey changes bearing. (3) *Holcocephala* changes its own bearing proportionally. (4) The range vectors are maintained parallel throughout the trajectory. (5) The change in prey direction has been successfully nulled. The target is caught in a few more milliseconds.

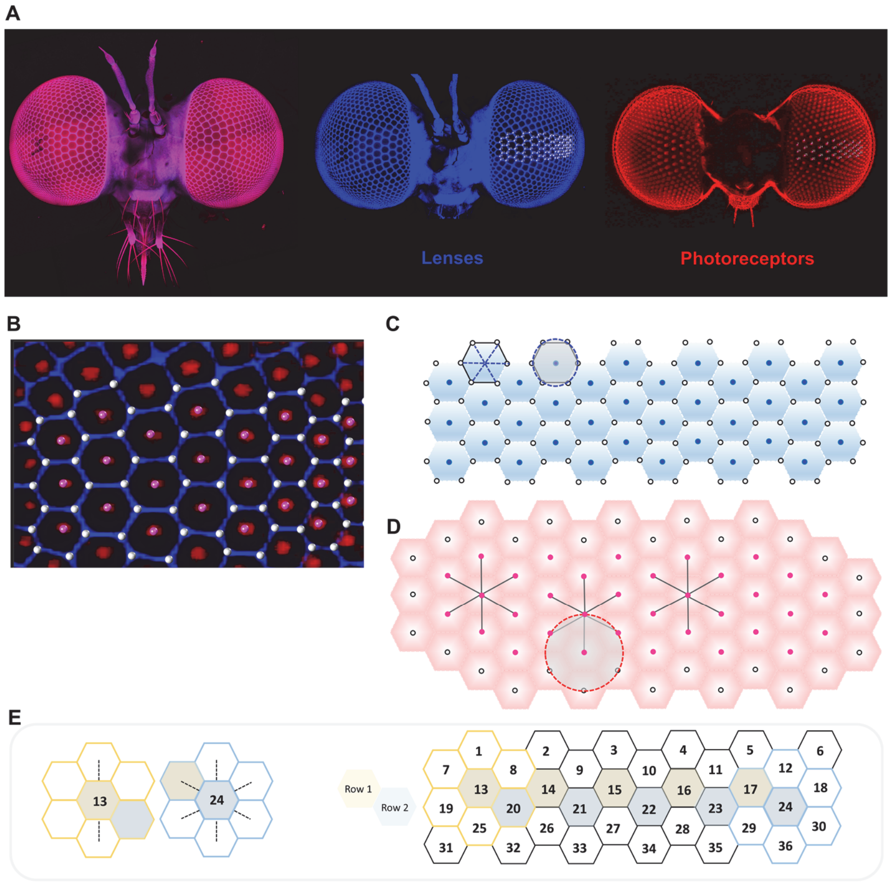

Figure S2. Related to Figure 4. Method for reconstructing interommatidial angles in *Holcocephala* through 2-photon microscopy. (**A**) Auto-fluorescence from a 3D stack of the *Holcocephala* head acquired with 2-photon microscopy. The markers placed on lenses and photoreceptors are displayed using data from a single colour channel with adjusted lookup tables to highlight features. (**B**) Close up image of a 3D stack with data from both red and blue channels displayed and overlaid with lens corner markers (white) and rhabdomere-tip markers (magenta). (**C**) The markers at the corners of each lens are used to calculate the lens diameter (average of 3 measurements, blue broken lines). Diagram showing how the centre of the lens (blue marker) is calculated as the centroid, or centre of mass, of the corner markers (i.e. average location of all 6 markers, blue dashed circle). (**D**) Diagram depicting how at the rhabdomere level, the ommatidia boundaries are not reliably detected. To reduce the error in the placement of the marker, the centroid method is employed: the centroid of the neighbouring rhabdomeres (red circle with dashed line) is used as the new rhabdomere location. In order to use this centroid method on all ommatidia, additional units need to be initially marked. The markers with recalculated locations are shown in magenta. This method yields "rosettes" formed by 1 centre ommatidium and 6 neighbouring ones (black lines), in which all the rhabdomere markers have been relocated. (**E**) Diagram showing how within each rosette, interommatidial angles are calculated as averages from either the vertical, or all neighbours. We placed sufficient markers to cover the entire fovea, and quantified 2 rows of 5 rosettes each.

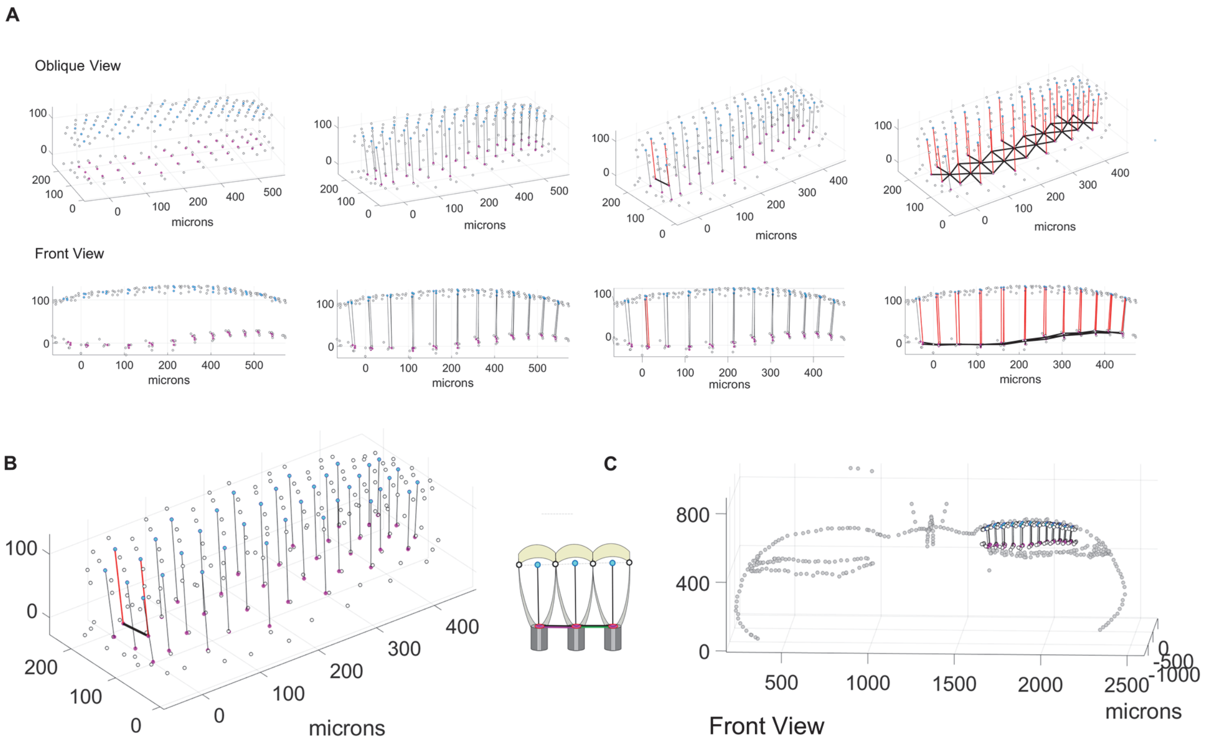

Figure S3. Related to Figure 4. Pipeline of the interommatidial angle reconstruction. (**A**) Figures showing the markers (as described in Extended Data Fig. S2B-E). In the cornea layer, white markers with a black edge are the lens corners and blue markers are the calculated lens centres. In the photoreceptor layer, white markers with a black edge are the original placement of the rhabdomere tips (as assessed by the experimenter) and pink markers are the recalculated location of the rhabdomere tips according to the centroid method. The visual axis of each ommatidium is given by the vertical line that joins the corresponding blue and pink markers. The interommatidial angle between any 2 units is calculated on the vertical plane that crosses the horizontal line (bold black) that joins them. This reduces the problem to 2D, and the angle between the two red vectors (the ommatidia axes) can be measured. (**B**) Magnified view showing that the relocation of the rhabdomere tips by the centroid method is minimal. (**C**) The reconstructed visual axes, within a digitized outline of the same *Holcocephala* head (grey markers).

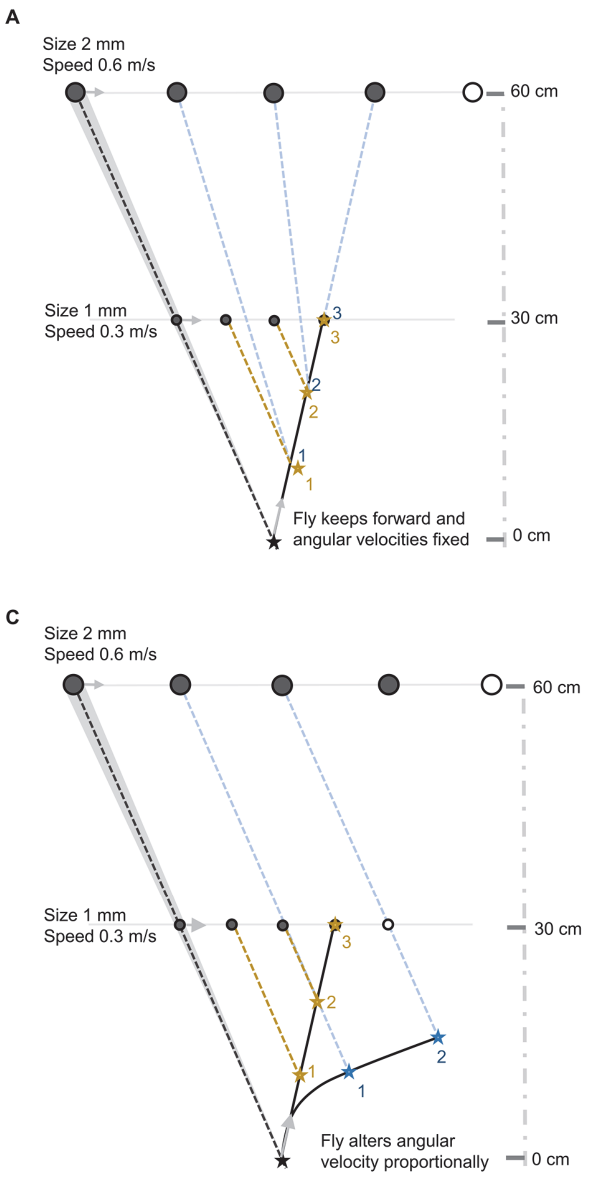

Figure S4. Related to Figure 2. Using the constant bearing angle strategy (CBA) during long range interception. (**A**) A 2 mm target is presented at 60 cm distance, but its size and distance are ambiguous to *Holcocephala* because this distance is outside of its depth perception range; it could be a slow moving small target that is close (1 mm bead) or it could be a fast moving large target that is far away (2 mm bead). *Holcocephala* starts with a speed appropriate to intercept the bead that is closer (1 mm bead). If this heading or velocity is not altered, the fly will "fall behind" and won't be able to intercept the actual target (the 2 mm bead). (**B**) Once airborne, the fly can compensate for the size/distance discrepancy by applying a forward acceleration such that the average velocity doubles, yielding a constant bearing angle. This action will keep the range vectors parallel, and assure an interception. (**C**) As an alternative, the fly can change its heading, this will maintain the range vector parallel, and eventually ensure a trajectory that does not require a forward speed as high as that of (B), but the rate of closure will drop, which means that the interception will take much longer.

Supplemental Experimental Procedures

Animals

Collection, identification and filming were undertaken within the regional Parks in York, Pennsylvania. Permits for site access to perform the research and for sample collection were obtained from the local parks authority. Two *Holcocephala* species, *H. fusca* and *H. abdominalis*, share a common range within the USA and are functionally cryptic species [S3]. We refer to this species as *H. fusca*. Within this field site we also encountered *H. calva*, which were excluded from the experiments.

Computer controlled bead mover: Fly teaser

Beads were presented to *Holcocephala* using a custom made plastic frame that housed a stepper motor and several pulleys. The whole ensemble is referred to as the "fly teaser" (**Figure 1**). The beads were attached to a taut fishing line which was wrapped around the stepper motor pulley and moved past the pulleys on the apparatus. This allowed the precise movement of beads at designated velocities and timings. The stepper motor was computer controlled and the whole apparatus was on a monopod that acted as a handle and allowed us to hold the fly teaser near a chosen perched fly. To simplify the problems of 3D interactions, we aimed to align the approaching direction of the target with the body axis of the fly. For more information on the stepper motor controller see [S4]. For the experiments in which several consecutive beads of different diameters were used, we are confident that flies did not have to examine several targets at once, because the time between the end of head movements related to a rejected bead and the start of head movement to the following bead was 680 ± 63 ms (mean ± SE, n = 8), while the delay between the end of a head movement and take-off was in all cases < 350 ms.

Acquisition of behavioural data

To improve the contrast of the camera images and help us digitize the recorded trajectories, white fabric was placed 1-3 metres horizontally away from the fly and used in all experiments as a backdrop. Since the flies contrast their target against the sky, and given that the cameras were placed at right angles to the fly anterior-posterior body axis, it is unlikely that the background interfered with the prey capture behaviour of the animals. Data were acquired with two synchronized Photron SA2 cameras (Photron Limited, Tokyo, Japan) running at 1000 fps. The system was calibrated using the Matlab toolbox by J.Y. Bouguet's laboratory (Caltech, http://www.vision.caltech.edu/bouguetj/calib_doc/) with numerous alterations. The position of the prey (or bead) and that of the predatory fly was digitized with custom written Matlab scripts. The resulting XYZ positions were fed to a fitting algorithm for trajectory reconstruction [S5]. ** For a detailed method on how to calibrate, digitize and smooth such data, request the Analysis Package.*

Analysing the aerial attacks

For each trajectory, we noted the frame at which the flies started to take off as the first visible movement of the wings. We then calculated the distance from fly to bead at that frame. We had noted during experimentation which bead size was presented. From such data, we calculated the subtended speed and size.

LOS range vector correlation

From the recorded trajectories 63 were chosen for vector correlation because they represented complete pursuits of beads with stable trajectories that ended in a visible catch. Four trajectories were chosen for vector correlation as the target changed direction in mid-flight and the fly continued to pursue and visibly catch the bead. Range vectors between the bead and the fly were calculated, in two different ways. **Figure 2** shows the signed difference between any range vector and the median vector, whereas **Figure S1** shows the neighbouring vector correlation.

To calculate the "lock on" distance we searched for the location of the fly at the time that the fly began a terminal deceleration whilst approaching the target. To find the maximum distance at which flies would initiate a lock on phase (**Figure 2C2**), we used a sigmoidal function with 4 coefficients ($f(x) = a+(b-a)/(1+10^{((c-x)d)})$). To quickly gather suitable staring point values for each of the four coefficients, the sigm_fit matlab function (Ohad Gal at Matlab file exchange) was used. The minimum value possible (a) was fixed to zero. The maximum value (b), inflexion point (c) and slope (d) were estimated by best fit. The values obtained with Sig_fit for each coefficient (0, 288, 290, 0.0037) were used as starting points in the Matlab curve fitting GUI, with all four coefficients free to be estimated by best fit. In this plot n = 86 because trajectories with such geometry acquired later in the study, with beads of different sizes, are also included. To calculate the average "lock on" speed we took the mean fly speed in the last 10% of the flight as the speed had plateaued in the majority of flights by this point.

Acquisition of microscopy data

We took advantage of the miniature size of *H. fusca* and imaged two whole mounted heads (2.9 mm wide x 0.82 mm deep). Tissue processing was done as in [S6], but with an additional first step that involved bleaching the samples as described in [S7, 8]. Imaging was completed using an Olympus XLSLPLN25XGMP objective, a Newport Spectra-Physics InSight® DS+™ laser at 810 nm, and a Bruker (Prairie Technologies) In Vivo Microscope using GFP and RFP detection channels. The voxel resolution was 1.75 µm in X and Y and 1.8 µm in Z. To obtain the semi-thin head sagittal sections and ultrathin eye cross-sections (for TEM imaging) shown in **Figure 3B-C**, the specimens were prepared as outlined in [S9].

We first identified the ommatidia that laid at the eye midline, where the Semper cells or the photoreceptor caps were still present, and then imaged the first neighbouring ommatidium in which the rhabdomeres were visible [SSee 10 for anatomical scheme]. In this manner we guaranteed that the measurements were taken at the tip of the rhabdomeres, which is important because fly rhabdomeres diverge quickly below this level to minimize crosstalk [S11]. After fitting the outline of each rhabdomere with an ellipse, we calculated the distances from the R7 rhabdomere to the others rhabdomeres. Photoreceptor 3 is excluded from the comparison because the neural superposition wiring dictates that such rhabdomere is not an immediate neighbour [S12]. We then calculated the mean from the three ommatidia with the closest rhabdomeres.

To obtain the focal length measurements, we employed the cornea drop method [Sas described in 13]. Briefly, a cornea was cut and any leftover cells from the retina were gently removed with a brush. The cornea was then suspended in a drop of saline and the magnification of the focused image produced was used to calculate the focal length. To find the longest focal length in a robust fashion, we averaged the values from the longest 3 focal lengths within our sample.

Analysis of microscopy data
Stacks from the 2-photon microscope were stitched and converted with Fiji [S14] so that it could be loaded into Vaa3D [S15]. The lookup tables were altered to increase the signal from the lenses or the rhabdomeres. Vaa3D was used to mark the location of the corner of the lenses and the location of the tip of the rhabdomere group in the ommatidia of interest. Marker files were named as Fly27, representative of a small fly, and Fly29, representative of a large fly among those collected. They thus provide us with a range of measurements reflective of the biology. In each eye, we used the cornea and the photoreceptor cell signals (**Figure S2A**) to place markers (**Figure S2B-E**) for the 5 rows of ommatidia next to the head suture (used as a landmark) and which always contained the largest lenses. From these parameters we reconstructed the lens diameter and visual axis of each ommatidium (**Figure 4**, **Figure S3**). We did not mark and reconstruct the rest of the eye because (*i*) the measurement error would have been bigger since at present the software used does not allow cutting the 3D stack in an arbitrary plane, and this would be necessary for accurate placement of markers as the curvature of the eye increases, and (*ii*) it would create a high work load, but provide no answers about the finest acuity obtained in this eye. ** For a detailed method on how to mark the lenses and the rhabdomeres, please request the Analysis Package.*

Large errors in marker placement were nulled by the centroid method employed (**Figure S2**). With regards to deformation in the sample, the clearing agent used produces minimal shrinkage [Ssee 6]. Moreover, although we noticed that in some samples the lower half of the photoreceptors exhibited a bend, their tips were always held in place firmly, aided by the robust proximity to the cornea and pseudocone. To calculate the error associated with using this method to measure lens diameters, we looked into the size differences between the corresponding ommatidia in the left and the right eyes (we did this for the 2 heads). Such difference is a composite of the natural variation in lens size between the two eyes and the variability of our measurement, and therefore it provides a conservative estimate of measurement error. We found that the difference in diameter between corresponding ommatidia was 2.1 ± 1.6 µm for fly 27 and 1.0 ± 1.1 µm for fly 29 (mean ± SD, n = 36). Thus, the measurements for each of ommatidium was not significantly different between the two eyes (p-values from two tailed, paired student t-test: fly 27 and 29: 0.22 and 0.19, respectively; df = 35. On average, the measurement error was 2% and 4% of the total lens diameter.

Ideally, we would have compared our measurement of interommatidial angles with those obtained with the pseudopupil method. However, the *Holcocephala* pseudopupil cannot be seen due to the dark pigmentation of the eye. Therefore, to estimate the error associated with our method we compared the interommatidial angles obtained for the corresponding ommatidia in both eyes (as explained above for the lens diameter). Instead of simply giving an average for the rosette (one rosette is composed of a single ommatidium and all its immediate neighbours), we kept the vertical and diagonal interommatidial angles separate because a paired t-test for all the measurements showed that the diagonal interommatidial angles are larger than vertical ones (mean ± SE for $\Delta\varphi$ vertical: 0.62° ± 0.04, and $\Delta\varphi$ diagonal: 1.03 ± 0.09; $p<0.001$). The vertical interommatidial angles were not affected by the location of the ommatidia (i.e. medial, centre or lateral). Therefore, we treated all measured vertical interommatidial angles as one population, with mean difference between the two eyes 0.19° ± 0.03 (mean ± SE, n = 20 ommatidia). To calculate the final vertical interommatidial angle range for each unit, we averaged the corresponding value for both head samples and then applied ± 0.19°. Visual inspection confirmed that there was a natural cut off between the interommatidial angle of the

5 centre ommatidia and the rest (**Figure 4D,** red lined hexagons). Those 5 interommatidial angles have a mean range between 0.21 to 0.59°.

Stereopsis range

The stereopsis range for this animal from interommatidial angles and from the inter acute-zone distance was calculated by employing the equation developed by [S16].

$$E_\infty = b^2/\tan(\Delta\varphi/2)$$

where b is the inter acute-zone distance and $\Delta\varphi$ is the interommatidial angle. This equation yields the maximal distance that the robber fly can distinguish from infinity by binocular triangulation to be ~ 26 cm.

Analysis package available on request

The package contains the following analysis guides, the Matlab scripts necessary to analyse the data and a sample data set for each:

1. *How to mark lenses and rhabdomeres*
2. *How to calibrate, digitize, smooth data.*

Supplemental References

S1. Fajen, B.R. (2013). Guiding locomotion in complex, dynamic environments. Front. Behav. Neurosci. *7*, 85.

S2. Mischiati, M., Lin, H.T., Herold, P., Imler, E., Olberg, R., and Leonardo, A. (2015). Internal models direct dragonfly interception steering. Nature *517*, 333-338.

S3. Fisher, E. (2006). Communication posted on http://bugguide.net/node/view/67616#68560. (Calif. Dept. of Food & Agriculture).

S4. Wardill, T.J., Knowles, K., Barlow, L., Tapia, G., Nordstrom, K., Olberg, R.M., and Gonzalez-Bellido, P.T. (2015). The killer fly hunger games: Target size and speed predict decision to pursuit. Brain Behav. Evol. *86*, 28-37.

S5. Dey, B., and Krishnaprasad, P.S. (2012). Trajectory smoothing as a linear optimal control problem. 50th Annual Allerton Conference on Communication, Control, and Computing (Allerton), 1490-1497.

S6. Gonzalez-Bellido, P.T., and Wardill, T.J. (2012). Labeling and confocal imaging of neurons in thick invertebrate tissue samples. Cold Spring Harb Protoc *2012*, 969-983.

S7. Stockl, A.L., and Heinze, S. (2015). A clearer view of the insect brain-combining bleaching with standard whole-mount immunocytochemistry allows confocal imaging of pigment-covered brain areas for 3D reconstruction. Front. Neuroanat. *9*, 121.

S8. Smolla, M., Ruchty, M., Nagel, M., and Kleineidam, C.J. (2014). Clearing pigmented insect cuticle to investigate small insects' organs in situ using confocal laser-scanning microscopy (CLSM). Arthropod Struct. Dev. *43*, 175-181.

S9. Gonzalez-Bellido, P.T., Wardill, T.J., and Juusola, M. (2011). Compound eyes and retinal information processing in miniature dipteran species match their specific ecological demands. Proc. Natl. Acad. Sci. U.S.A. *108*, 4224-4229.

S10. Katz, B., and Minke, B. (2009). *Drosophila* photoreceptors and signaling mechanisms. Front. Cell Neurosci. *3*, 2.

S11. Wijngaard, W., and Stavenga, D.G. (1975). On optical crosstalk between fly rhabdomeres. Biol. Cybern. *18*, 61-67.

S12. Stavenga, D.G. (1975). The neural superposition eye and its optical demands. J. Comp. Physiol. *102*, 297-304.

S13. Stowasser, A., Rapaport, A., Layne, J.E., Morgan, R.C., and Buschbeck, E.K. (2010). Biological bifocal lenses with image separation. Curr. Biol. *20*, 1482-1486.

S14. Schindelin, J., Arganda-Carreras, I., Frise, E., Kaynig, V., Longair, M., Pietzsch, T., Preibisch, S., Rueden, C., Saalfeld, S., Schmid, B., et al. (2012). Fiji: an open-source platform for biological-image analysis. Nat. Methods *9*, 676-682.

S15. Peng, H., Bria, A., Zhou, Z., Iannello, G., and Long, F. (2014). Extensible visualization and analysis for multidimensional images using Vaa3D. Nat. Protoc. *9*, 193-208.

S16. Burkhardt, D., Darnhofer-Demar, B., and Fischer, K. (1973). Zum binokularen entfernungssehen der Insekten. 1. Die struktur des sehraums von synsekten. J. Comp. Physiol. A Neuroethol. Sens. Neural. Behav. Physiol. *87*.
