# Supplementary material for: A Novel Interception Strategy in a Miniature Robber Fly with Extreme Visual Acuity
Source: Curr Biol. 2017 Mar 20;27(6):854–9. doi: 10.1016/j.cub.2017.01.050 (PMC5364399; doi:10.1016/j.cub.2017.01.050)
Supplement: Document S2. Article plus Supplemental Information [file mmc5.pdf]

Current Biology

Report

A Novel Interception Strategy in a Miniature Robber Fly with Extreme Visual Acuity

Graphical Abstract

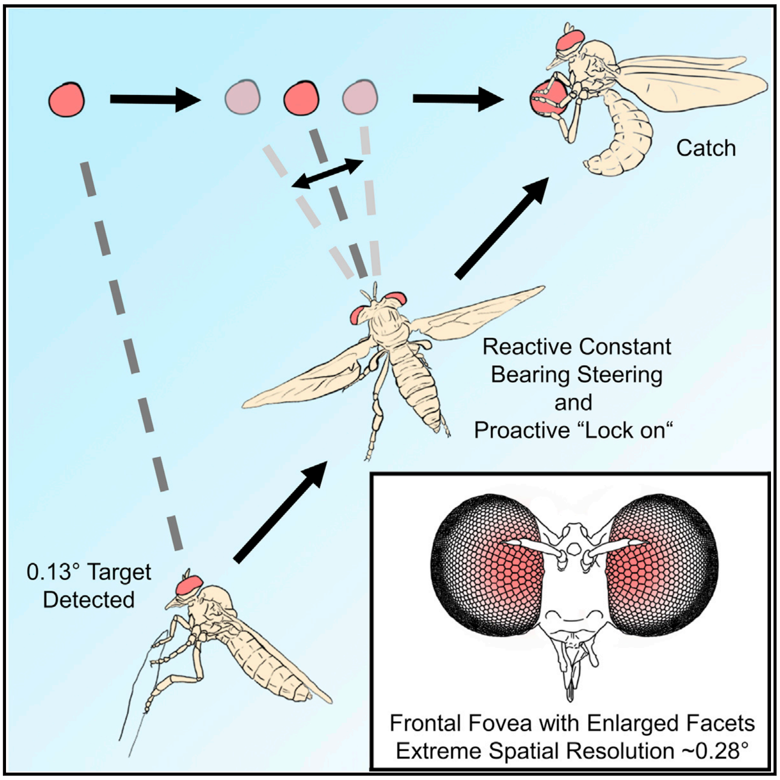

Highlights

- *Holcocephala fusca* robber flies capture prey with a constant bearing angle strategy
- The approach is proactively altered to "lock on" on to prey within 29 cm distance
- The retina spatial resolution is 0.28°, but the object detection threshold is 0.13°
- The fly's stereopsis range is estimated to be ~26 cm

Authors

Trevor J. Wardill, Samuel T. Fabian, Ann C. Pettigrew, Doekele G. Stavenga, Karin Nordström, Paloma T. Gonzalez-Bellido

Correspondence

tjw79@cam.ac.uk (T.J.W.),
ptg25@cam.ac.uk (P.T.G.-B.)

In Brief

Robber flies are aerial predators. Here, Wardill, Fabian, et al. show that the tiny robber fly *Holcocephala fusca* attacks a detected prey reactively but proactively changes its speed and direction when the prey is within 29 cm. They also show that the very small object detection threshold of 0.13° is supported by a striking visual fovea.

Wardill et al., 2017, Current Biology *27*, 854–859
March 20, 2017 © 2017 The Author(s). Published by Elsevier Ltd.
http://dx.doi.org/10.1016/j.cub.2017.01.050

Current Biology
Report

A Novel Interception Strategy in a Miniature Robber Fly with Extreme Visual Acuity

Trevor J. Wardill,[1,2,6,*] Samuel T. Fabian,[1,6] Ann C. Pettigrew,[3] Doekele G. Stavenga,[4] Karin Nordström,[5] and Paloma T. Gonzalez-Bellido[1,2,7,*]
[1]Department of Physiology, Development and Neuroscience, University of Cambridge, Downing Street, Cambridge CB3 2EG, UK
[2]Eugene Bell Center for Regenerative Biology and Tissue Engineering, MBL, 7 MBL Street, Woods Hole, MA 02543, USA
[3]Leader Heights Animal Hospital, 199 Leaders Heights Road, York, PA 17402, USA
[4]Computational Physics, Zernike Institute for Advanced Materials, University of Groningen, Groningen 9747 AG, the Netherlands
[5]Centre for Neuroscience, Flinders University, GPO Box 2100, Adelaide, SA 5001, Australia
[6]Co-first author
[7]Lead Contact
*Correspondence: tjw79@cam.ac.uk (T.J.W.), ptg25@cam.ac.uk (P.T.G.-B.)
http://dx.doi.org/10.1016/j.cub.2017.01.050

SUMMARY

Our visual system allows us to rapidly identify and intercept a moving object. When this object is far away, we base the trajectory on the target's location relative to an external frame of reference [1]. This process forms the basis for the constant bearing angle (CBA) model, a reactive strategy that ensures interception since the bearing angle, formed between the line joining pursuer and target (called the range vector) and an external reference line, is held constant [2–4]. The CBA model may be a fundamental and widespread strategy, as it is also known to explain the interception trajectories of bats and fish [5, 6]. Here, we show that the aerial attack of the tiny robber fly *Holcocephala fusca* is consistent with the CBA model. In addition, *Holcocephala fusca* displays a novel proactive strategy, termed "lock-on" phase, embedded with the later part of the flight. We found the object detection threshold for this species to be 0.13°, enabled by an extremely specialized, forward pointing fovea (~5 ommatidia wide, interommatidial angle $\Delta\varphi$ = 0.28°, photoreceptor acceptance angle $\Delta\rho$ = 0.27°). This study furthers our understanding of the accurate performance that a miniature brain can achieve in highly demanding sensorimotor tasks and suggests the presence of equivalent mechanisms for target interception across a wide range of taxa.

RESULTS

Aerial Attack Strategy

In our study of the aerial hunts of the robber fly *Holcocephala* (Figure 1), we considered whether its behavior is consistent with the constant bearing angle (CBA) model (Figure S1). We tested this on flies in their natural habitat by presenting a range of beads (diameter 1.3, 2.9, and 3.9 mm) on a fishing line, whose speed was controlled by a stepper motor (Figure 1B; Supplemental Experimental Procedures). We recorded the flies' behavior with two high-speed video cameras and reconstructed their flight trajectory in three dimensions. Consistent with the CBA model, we found that when pursuing a bead moving at constant speed, the range vectors were close to being parallel across most of the trajectory: for 80% of the flight time, the absolute difference between each range vector and the trajectory median range vector was on average less than 3° (Figures 2A1–2A3; n = 63 attacks to a 1.3 mm bead). By applying proportional navigation, the guidance law associated with the CBA model [4, 7], a pursuer can control the necessary steering command to null any change in the velocity of the target, thereby keeping the range vectors parallel and actively maintaining the CBA (Figures S1G and S1H). We tested the CBA mechanism of *Holcocephala* by decelerating or reversing the bead during the attack (Movie S1). We found that *Holcocephala* compensates for bead trajectory changes and actively keeps the range vectors parallel (Figures 2B1–2B3; n = 4 attacks to a 1.3 mm bead), consistent with achieving a CBA through proportional navigation.

One surprising finding was that the latter part of *Holcocephala*'s pursuing trajectory was distinctly curved. This was most apparent when the targeted bead traveled toward the front of the animal and *Holcocephala* took off with a "head-on" collision course but ultimately intercepted the bead while flying backward (Figure 2C1; seen in 22 of the 63 analyzed trajectories toward the 1.3 mm bead). Under a CBA strategy, compensatory flight alterations are necessary if the prey alters its velocity (direction, speed, or both), but since the targets were presented with constant velocity, the fly's change in direction was not elicited by the target. Could *Holcocephala* simply have miscalculated the heading necessary for a straight interception or perhaps failed to attain the speed necessary to intercept with such a heading? This is unlikely, as extending the initial *Holcocephala* trajectory along the velocity vector attained just before the change in heading (Figure 2C1, turquoise broken line) shows that *Holcocephala* would have been very near the interception point (minimal distance 2.9 ± 0.4 cm, mean ± SE, n = 22). Presumably this small error could have been easily corrected during the rest of the attack. Nonetheless, the observed curved trajectory

 © 2017 The Author(s). Published by Elsevier Ltd.
This is an open access article under the CC BY license (http://creativecommons.org/licenses/by/4.0/).

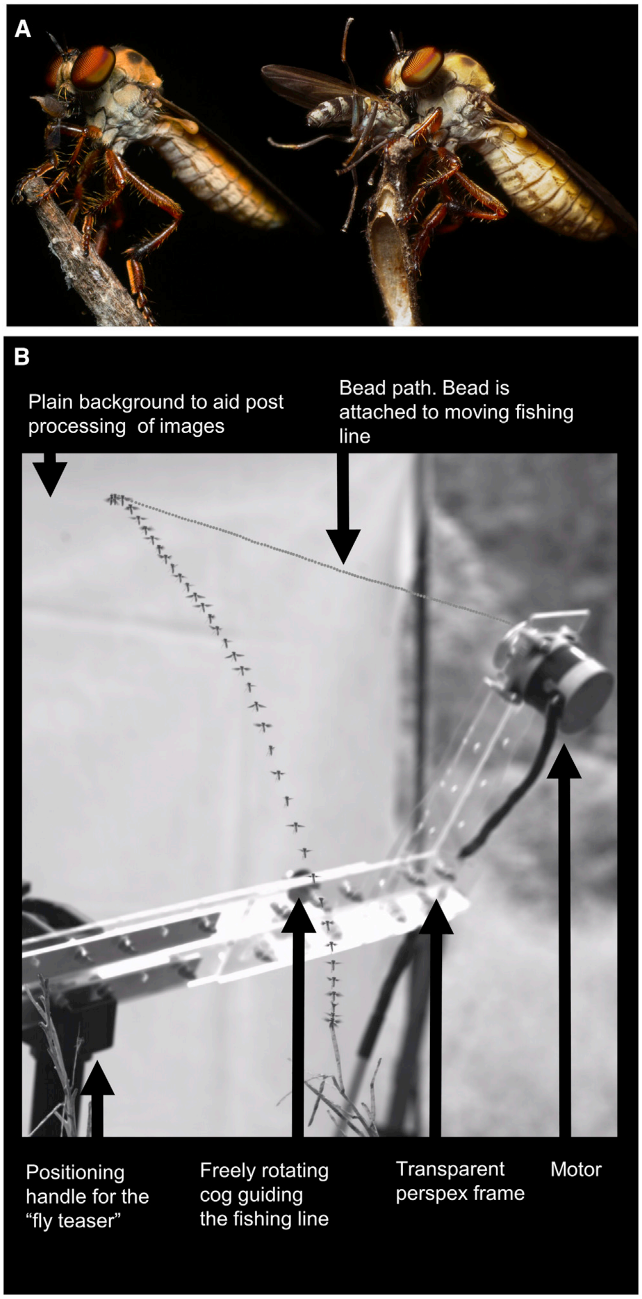

Figure 1. *Holcocephala* with Prey Items and Test of *Holcocephala*'s Predatory Behavior

(A) Two examples of *Holcocephala* feeding on prey caught mid-air with the smallest and largest prey that we observed.

(B) The "fly teaser" ensemble, used to entice flies to attack artificial targets in their natural environment, which allowed for controlled stimulus parameters, such as size, distance, and speed. Overlay: simultaneous positions of the fly and bead throughout the trajectory.

added to the total interception time on average 132 ± 7 ms (mean ± SE) until contact, which is a substantial 27% ± 1% of the total flight time (mean ± SE, n = 22).

To understand what induced the change in heading, we first looked at its timing. Our analysis showed that the change in heading was accompanied by a deceleration. We therefore took the first time point in the trajectory where a deceleration was detected as an objective measure for the start of the change in heading. By fitting the data with a sigmoidal curve (trajectories to beads of all three sizes in which a clear deceleration was detected were included; n = 86), we found that independent of the starting distance, the maximum distance between target and fly at which the change occurred was 29 ± 4 cm (95% confidence bounds; R^2 adjusted = 0.73; Figure 2C2). We also found that shortly after the change in heading, *Holcocephala* fixed its forward velocity vector slightly above that of the bead (Figure 2D). Our results reflect a "lock-on" process, initiated by information that becomes available once the fly is within ~29 cm of the target. Here, we use "lock-on" to refer to the phase during which the fly has a new heading and the speed is fixed to a value slightly higher than that of the prey. The different mechanisms that could underlie this behavior are addressed in the discussion.

Minimum Behavioral Discrimination of a Moving Target—Single Object Threshold—and Acuity Parameters of *Holcocephala*'s Fovea

The five longest target detection distances in our CBA experiments that lead to a successful catch of the 1.3 mm bead were > 53 cm (for example, Movie S2), and at such distances the bead subtended no more than 0.12°–0.14° on the retina. Therefore, the minimum single object threshold resolved by *Holcocephala*'s visual system must be as small as ~0.13°. The *Holcocephala* eye has an ommatidial lattice with an excessive gradient in facet size, which indicates the presence of a fovea with an extreme degree of functional regionalization. We therefore investigated the internal anatomy, to further elucidate the retinal adaptations that provide the necessary spatial visual performance driving the fly's pursuit behavior. Sectioning the *Holcocephala* eye revealed a reduced curvature of the frontal cornea as well as flattening of the basement membrane. The extremely enlarged frontal ommatidia have facet lenses with extended focal lengths that focus incident light into unusually slender rhabdomeres (Figure 3). These specializations are known to optimize the spatial resolution of fly eyes, thus creating an area of high acuity, a fovea [8].

Crucial measures for the spatial acuity of a compound eye are the photoreceptor acceptance angle, $\Delta\rho$, and the interommatidial angle, $\Delta\varphi$, the angle between the optical axes of neighboring ommatidia. The photoreceptor acceptance angle can be estimated from the ratio of the rhabdomere diameter (D_r) and the facet lens's focal length (f). Anatomical measurements yielded $D_r = 0.92 \pm 0.13$ μm (Figure 3C). Using the hanging drop method with cleaned corneas in the eye region with the largest facet lenses (Figure 3D), the focal length was found to be $f = 190 \pm 4$ μm, hence yielding a very small photoreceptor acceptance angle: $\Delta\rho = 0.28 \pm 0.04°$. Measurement of the interommatidial angle with the preferred pseudopupil method [9] was problematic due to *Holcocephala*'s dense eye pigmentation, and therefore we considered the unique property of fly eyes where sets of six photoreceptors located in six adjacent ommatidia pool their signals in one cartridge of the lamina, the first optical ganglion below the retina. This neural superposition principle dictates that the interphotoreceptor angle, the angle between the

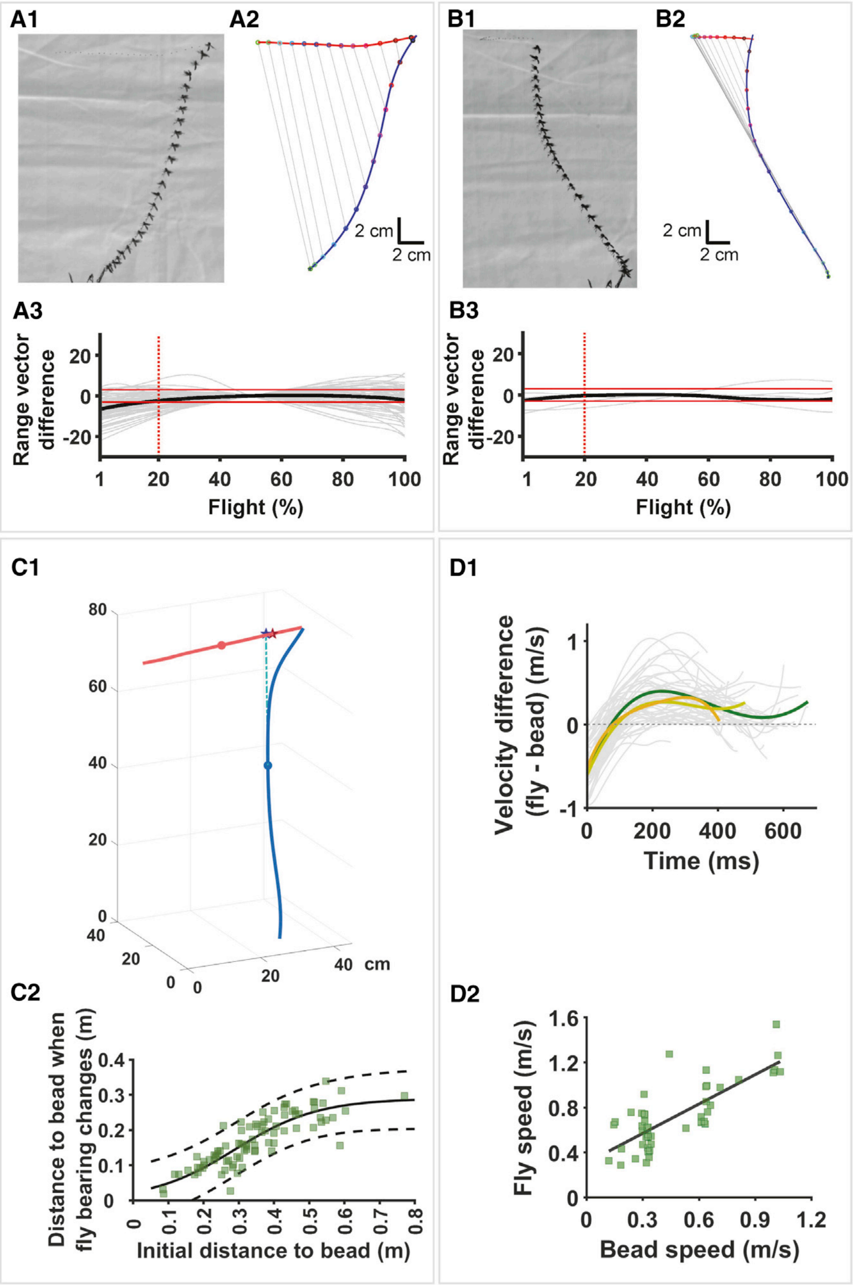

Figure 2. Geometry and Timing of the *Holcocephala* Aerial Attack

(A1) *Holcocephala* flight trajectory toward a target moving at constant speed.

(A2) 3D reconstructed trajectory of the flight course (blue curve) showing nearly parallel range vectors of decreasing length (target trajectory: red curve).

(A3) The difference in direction (in degrees) between any one range vector (the line joining predator and prey at each frame) and the median range vector for the trajectory plotted for all trials in which *Holcocephala* chased a target moving at constant speed (n = 63; solid red lines = −3° and +3°; dotted red line: 20% of flight time elapsed; see also Figure S1).

(B1 and B2) Flight trajectory when the presented bead changes velocity and completely reverses direction, during which *Holcocephala* maneuvers to keep the range vector parallel (see also Movie S1).

(B3) During bead reversal presentations, the difference between the range vectors and the median vector stays close to zero (n = 4).

(C1) Trajectory that would have resulted in a head-on collision interception (cyan dashed line), but before the collision *Holcocephala* arched backward (blue line).

(C2) Distance to target when the change in heading occurs (black line: four-parameter sigmoidal fit; adjusted r^2 = 0.73; 95% confidence bounds shown by broken lines; n = 86).

(D1) The difference in velocity between fly and bead. After the initial phase, the flies stop accelerating and keep their speed at a value that is slightly higher than that of the bead; this behavior is independent of attack duration (average short, medium, and long trajectories shown in short orange, medium lime, and long green lines, respectively).

(D2) Fly speed as a function of bead speed. The average velocity during the lock-on phase is correlated with that of the bead (adjusted r^2 = 0.6; for all D plots, n = 51).

See also Figure S4 and Movie S2.

visual axes of the photoreceptors within one and the same ommatidium, equals the local interommatidial angle. Yet, in a detailed study on a number of fly species, Pick [10] demonstrated that the interphotoreceptor angle is actually ~20% larger than the interommatidial angle. The interphotoreceptor angle in an ommatidium equals the ratio of the distance between adjacent rhabdomeres and the focal length of the facet lens. From ultrathin cross-sections of the eye region with the largest facet lenses (see Supplemental Experimental Procedures), we found that the interrhabdomere distance was D_i = 1.15 ± 0.16 μm, thus yielding the interphotoreceptor angle D_i/f = 0.35° ± 0.05°, or, applying Pick's [10] correction, the interommatidial angle becomes $\Delta\varphi$ = 0.28° ± 0.04°.

Arrangement of Interommatidial Angles and Direction of Visual Axes in the Fovea

To understand the distribution of the visual axes across the fovea, we acquired two-photon microscopy images of the fovea region, yielding 3D anatomical stacks of the eye fovea's anatomy (Movie S3). The fovea appeared to be ~5 ommatidia wide, with diameter of the facet lenses 70–78 μm (Figures 4A and 4B) and interommatidial angles $\Delta\varphi$ = 0.40° ± 0.19° (Figures 4C and 4D; Figures S2 and S3; Supplemental Experimental Procedures). The interommatidial angles deduced above ($\Delta\varphi$ = 0.28° ± 0.04°) are within this range. The two-photon microscopy images further demonstrated that the central ommatidia of the acute zones in the two eyes have virtually parallel visual axes, meaning that *Holcocephala* has a binocular view of the world (Figure 4E). In summary, the behavioral performance that we measured (>53 cm interception distances and ~0.13° object threshold) is supported

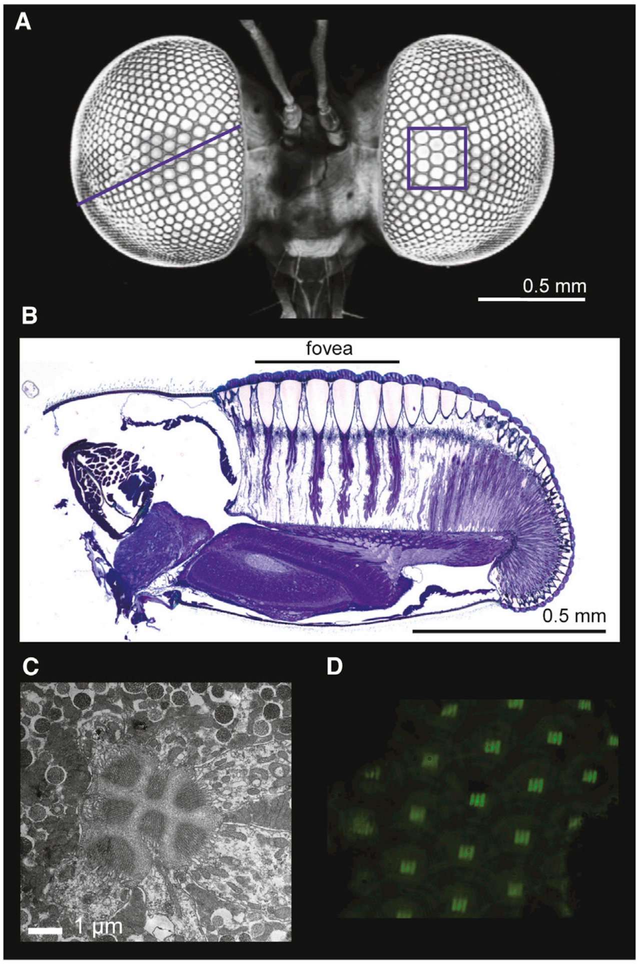

Figure 3. Structural Specializations of the *Holcocephala* Eye

(A) Two-photon image of the *Holcocephala* head showing the enlarged frontal facets. The line and box mark the locations of the oblique section shown in (B) and the cross-section shown in (C), respectively.

(B) Oblique eye section showing the acute zone with enlarged sizes and focal lengths of the facet lenses, as well as a flat cornea and basement membrane.

(C) Cross-section of an ommatidium in the acute zone showing the rhabdomeres with tip diameter ~0.9 µm.

(D) Image of a grating pattern created by an isolated cornea with the hanging drop method, which allowed calculation of the focal length (*f*).

by the fine spatial resolution (0.28°) provided by the specialized fovea.

DISCUSSION

We have investigated the predatory attack of the robber fly *Holcocephala fusca* and found that this species generates an interception course using a constant bearing angle strategy and applying maximum acceleration to quickly get closer to the prey. It is not surprising that *Holcocephala* utilizes a CBA strategy during most of its flight, as this reactive strategy enables compensation for (1) unexpected changes in the target's velocity and (2) uncertainties about the perceived location, size, and speed of the target when absolute depth cues are absent (Figure S4). Even humans, who arguably have higher computational brain power, rely on a CBA to solve similar tasks [1]. This indicates that the CBA strategy is a robust way to intercept targets when sensory information is limited, independent of the processing power available. To further confirm the use of proportional navigation, future studies will need to embed this guidance law in a control architecture that can match entire trajectories. It is also of importance to note that the use of a CBA reactive strategy does not exclude other guiding principles from being applied. For example, during their interception flights, dragonflies use both reactive and proactive motor commands [11]. It is therefore of interest that once *Holcocephala* is within ~29 cm of the target, it implements a heading and speed change. The presence of such a lock-on phase has not yet been described in any other flying animal. Although the lock-on strategy extends the total flight time (Figure 2), lowering the final flight speed needed for interception and extending the time over which *Holcocephala* may catch the prey is likely a highly effective adaptive behavior, consistent with the priority to ensure highest success rates in the face of sensorimotor delays and errors. The resulting strategy is similar to that of a baton pass in a relay race: a pass between two runners with similar direction and velocity is more likely to be successful than one between two runners passing each other in opposite directions.

What mechanisms could explain the trigger of the lock-on phase? It is possible that the lock-on phase is driven by invariant properties of the image, and not by actual distance estimation. For example, the escape responses of locusts, frogs, fruit flies, and crabs to a looming stimulus occur after the target reaches a certain angular size threshold [12–15]. However, when testing the lock-on phase by offering beads with different sizes, we found that at the moment the lock-on phase was initiated, the angular size of the target varied significantly (large bead: 1.23° ± 0.66°, medium bead: 0.81° ± 0.22°, small bead: 0.52° ± 0.67°, mean ± SD; n = 14, 9, and 63, respectively; p = 0.001 ANOVA). Therefore, the trigger for the lock-on phase is unlikely to be a specific subtended angular size. Nevertheless, similar to flies initiating deceleration prior to landing [16], *Holcocephala* may have used as the trigger the angular size of the object over its rate of expansion. This ratio, often referred to as optical tau [17], provides an estimate for time to contact and can be used as a threshold for initiation of a motor command with appropriate timing [18]. However, the *Holcocephala* attack violates two conditions that must be met for optical tau to be reliable: constant approach speed and symmetrical head-on approach [16]. Moreover, optical tau obtained from a target subtending a small size is unreliable [19] because the calculation depends on the perceived expansion rate. At the maximum distance at which the lock-on phase is initiated (~29 cm), the 1.3 mm bead subtends ~0.26°. With a foveal interommatidial angle of 0.28°, the bead will be detected by at most two ommatidia. Although it is conceivable that the rate of change in light intensity falling on a single ommatidium may act as an "expansion" parameter, to our knowledge, animals performing aerial pursuits do not exploit the contrast change in a single light detector as a reliable cue to calculate time to contact. Likewise, by translating or pivoting, an array of parallel sensors can provide depth and range information [20], and a similar mechanism cannot be excluded without further analysis.

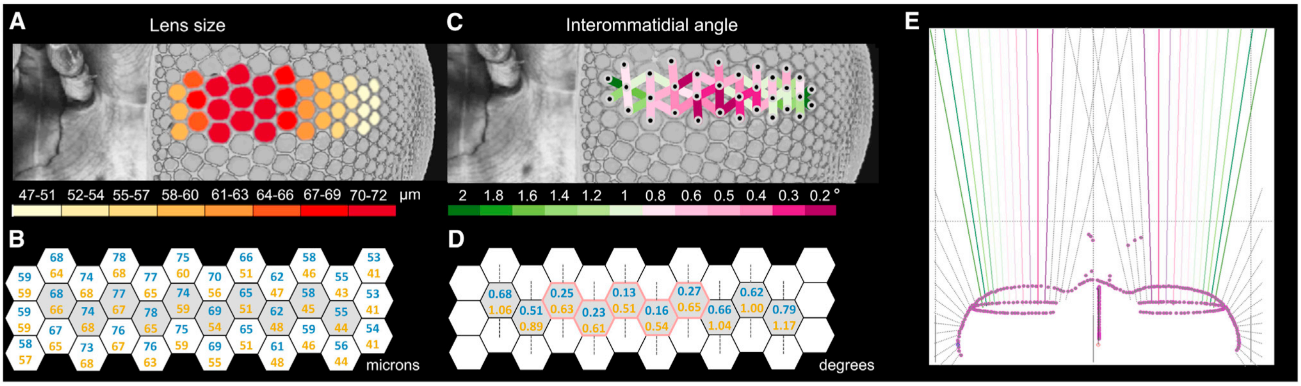

Figure 4. Optical Characteristics of the *Holcocephala* Fovea

(A) Distribution of the average diameters of the foveal facet lenses (values in look-up table in µm; mean of n = 4).

(B) Lens diameter (in µm) for a large fly (blue) and a small fly (yellow).

(C) Interommatidial angles derived from two-photon microscopy images (in degrees; n = 4); see also Figures S2 and S3.

(D) Range of vertical interommatidial angles (in degrees; n = 4); blue and yellow values are mean ± 0.19°.

(E) Ommatidial axes (colored lines) from a *Holcocephala* sample. The dark pink lines denote the centers of the acute zones where the axes of the central ommatidia are virtually parallel; the rest of the ommatidial axes diverge progressively. The dotted gray lines, indicating the additional ommatidial axes outside the fovea, were not measured but are added to heuristically illustrate the whole visual field.

See also Movie S3.

Alternatively, distance estimation via stereopsis could underlie the trigger of the lock-on phase in *Holcocephala*. Stereopsis is the reconstruction of depth from the disparity in the two ocular images due to the distance between the eyes [21]. If the visual fields of both eyes overlap sufficiently, the stereopsis range is solely dependent on the resolution of the retina and the distance between the forward-facing foveas [22]. Indeed, short range stereopsis has been demonstrated in mantids [23–25], and extended stereopsis has been predicted in mantid and dragonfly larvae with stereopsis ranges 46 and 26 cm, respectively [26]. *Holcocephala* also has a binocular field of view (Figure 4E). Given that the largest *Holcocephala* sample in our collection has an inter-fovea distance of 1.3 mm, with an interommatidial angle $\Delta\varphi = 0.28^\circ$ the limit of the stereopsis range is 26 cm. Given the small photoreceptor angle of $\Delta\rho = 0.27^\circ$, it may be feasible that *Holcocephala* uses depth cues provided by stereopsis to trigger the lock-on phase. Stereopsis at such range would require that the target be foveated, and it is possible that the head movements exhibited by *Holcocephala* prior to launching an attack serve such purpose. Whether or not *Holcocephala* uses time-to-contact acquired from monocular or binocular cues, the process bears parallels to the strategies employed by humans. For instance, when carrying out a long range interception, humans use both optical tau [27] and binocular cues [28] to improve the performance of reaching and grasping movements, and such prehensile movements form the second phase of a given task.

The existence of a localized area of high resolution in compound eyes, also called acute zone, dorsal zone, or love spot, is well documented among insect species that depend on target tracking for survival or mating [29], but the *Holcocephala* fovea clearly provides an extreme case. For example, the ~20 foveal ommatidia occupy ~20% of the eye volume (Figure 3) and span ~0.1% of the eye's visual space (angular range < 4.5°; Figure 4). In summary, our behavioral results of *Holcocephala* and the anatomical and optical data of its eyes demonstrate the extremely specialized visual capacities of a very small robber fly. Our findings may provide the basis of bioinspired guidance systems in miniature, aerial and autonomous vehicles, where maximum performance with minimum size is highly desirable.

SUPPLEMENTAL INFORMATION

Supplemental Information includes Supplemental Experimental Procedures, four figures, and three movies and can be found with this article online at http://dx.doi.org/10.1016/j.cub.2017.01.050.

A video abstract is available at http://dx.doi.org/10.1016/j.cub.2017.01.050#mmc6.

AUTHOR CONTRIBUTIONS

A.C.P. searched for and located the animals and reported their biology ahead of the experimental tests. T.J.W. developed the methodology for the behavioral experiments and two-photon imaging. T.J.W. and P.T.G.-B. designed the behavioral experiments, and T.J.W., S.T.F., and P.T.G.-B. conducted them. S.T.F. analyzed all other behavioral data. T.J.W., P.T.G.-B., and S.T.F. acquired the microscopy data. S.T.F. and P.T.G.-B. analyzed it. D.G.S. provided assistance in the optical calculations. K.N. collaborated in analyzing the results. All authors contributed to critical discussions and the writing of the manuscript.

ACKNOWLEDGMENTS

This work was funded by the Air Force Office of Scientific Research (FA9550-15-1-0188 to P.T.G.-B. and K.N. and FA9550-15-1-0068 to D.G.S.), an Isaac Newton Trust/Wellcome Trust ISSF/University of Cambridge Joint Research Grant (097814/Z/11/Z) to P.T.G.-B., a Biotechnology and Biological Sciences Research Council David Phillips Fellowship (BBSRC, BB/L024667/1) to T.J.W., a Royal Society International Exchange Scheme grant to P.T.G.-B. (75166), a Swedish Research Council grant (2012-4740) to K.N., and a Shared Equipment Grant from the School of Biological Sciences (University of Cambridge, RG70368). The TEM images were obtained at the CAIC in Cambridge. We are thankful to Elke K. Buschbeck for providing technical and practical advice and for her generosity sharing her equipment to obtain the focal length measurements. We thank Michael Land, Rob Olberg, Claude Desplan, and

Tom Cronin for helpful comments on the manuscript; Francis Velasquez for his continuous logistic support; staff at Nixon Park for supporting fieldwork; York College for kindly providing us access to their facilities; and Brian Jones for laser cutting assistance. We are thankful to Mary Sumner for fly tracking and data analysis support.

Received: July 31, 2016
Revised: December 16, 2016
Accepted: January 24, 2017
Published: March 9, 2017

REFERENCES

1. Fink, P.W., Foo, P.S., and Warren, W.H. (2009). Catching fly balls in virtual reality: a critical test of the outfielder problem. J. Vis. *9*, 1–8.
2. Diaz, G.J., Phillips, F., and Fajen, B.R. (2009). Intercepting moving targets: a little foresight helps a lot. Exp. Brain Res. *195*, 345–360.
3. Fajen, B.R. (2013). Guiding locomotion in complex, dynamic environments. Front. Behav. Neurosci. *7*, 85.
4. Gonzalez-Bellido, P.T., Fabian, S.T., and Nordström, K. (2016). Target detection in insects: optical, neural and behavioral optimizations. Curr. Opin. Neurobiol. *41*, 122–128.
5. Ghose, K., Horiuchi, T.K., Krishnaprasad, P.S., and Moss, C.F. (2006). Echolocating bats use a nearly time-optimal strategy to intercept prey. PLoS Biol. *4*, e108.
6. Lanchester, B.S., and Mark, R.F. (1975). Pursuit and prediction in the tracking of moving food by a teleost fish (*Acanthaluteres spilomelanurus*). J. Exp. Biol. *63*, 627–645.
7. Peppas, D. (1992). A computer analysis of proportional navigation and command to line of sight of a command guided missile for a point defence system. MA dissertation (Naval Postgraduate School).
8. Land, M.F., and Nilsson, D.E. (2002). Animal Eyes (Oxford University Press).
9. Stavenga, D.G. (1979). Pseudopupils of compound eyes. In Handbook of Sensory Physiology, Volume VII/6A, H. Autrum, ed. (Springer), pp. 357–439.
10. Pick, B. (1977). Specific misalignments of rhabdomere visual axes in the neural superposition eye of dipteran flies. Biol. Cybern. *26*, 215–224.
11. Mischiati, M., Lin, H.T., Herold, P., Imler, E., Olberg, R., and Leonardo, A. (2015). Internal models direct dragonfly interception steering. Nature *517*, 333–338.
12. Fotowat, H., Fayyazuddin, A., Bellen, H.J., and Gabbiani, F. (2009). A novel neuronal pathway for visually guided escape in *Drosophila melanogaster*. J. Neurophysiol. *102*, 875–885.
13. Gabbiani, F., Krapp, H.G., and Laurent, G. (1999). Computation of object approach by a wide-field, motion-sensitive neuron. J. Neurosci. *19*, 1122–1141.
14. Nakagawa, H., and Nishioka, R. (2010). The angular threshold for frog collision avoidance behavior changes depending on not only the stimulus location but also the behavioral strategy. In Brain-Inspired Information Technology, A. Hanazawa, T. Miki, and K. Horio, eds. (Springer), pp. 109–113.
15. Oliva, D., and Tomsic, D. (2014). Computation of object approach by a system of visual motion-sensitive neurons in the crab *Neohelice*. J. Neurophysiol. *112*, 1477–1490.
16. van Breugel, F., and Dickinson, M.H. (2012). The visual control of landing and obstacle avoidance in the fruit fly *Drosophila melanogaster*. J. Exp. Biol. *215*, 1783–1798.
17. Lee, D.N. (1976). A theory of visual control of braking based on information about time-to-collision. Perception *5*, 437–459.
18. Baird, E., Boeddeker, N., Ibbotson, M.R., and Srinivasan, M.V. (2013). A universal strategy for visually guided landing. Proc. Natl. Acad. Sci. USA *110*, 18686–18691.
19. Gray, R., and Regan, D. (1998). Accuracy of estimating time to collision using binocular and monocular information. Vision Res. *38*, 499–512.
20. Chahl, J.S. (2014). Range and egomotion estimation from compound photodetector arrays with parallel optical axis using optical flow techniques. Appl. Opt. *53*, 368–375.
21. Ponce, C.R., and Born, R.T. (2008). Stereopsis. Curr. Biol. *18*, R845–R850.
22. Burkhardt, D., Darnhofer-Demar, B., and Fischer, K. (1973). Zum binokularen Entfernungssehen der Insekten. 1. Die Struktur des Sehraums von Synsekten. J. Comp. Physiol. *87*, 165–188.
23. Nityananda, V., Tarawneh, G., Rosner, R., Nicolas, J., Crichton, S., and Read, J. (2016). Insect stereopsis demonstrated using a 3D insect cinema. Sci. Rep. *6*, 18718.
24. Rossel, S. (1983). Binocular stereopsis in an insect. Nature *302*, 821–822.
25. Rossel, S. (1996). Binocular vision in insects: how mantids solve the correspondence problem. Proc. Natl. Acad. Sci. USA *93*, 13229–13232.
26. Schwind, R. (1989). Size and distance perception in compound eyes. In Facets of Vision, D.G. Stavenga, and R.C. Hardie, eds. (Springer), pp. 425–444.
27. Savelsbergh, G.J., Whiting, H.T., and Bootsma, R.J. (1991). Grasping tau. J. Exp. Psychol. Hum. Percept. Perform. *17*, 315–322.
28. Servos, P., Goodale, M.A., and Jakobson, L.S. (1992). The role of binocular vision in prehension: a kinematic analysis. Vision Res. *32*, 1513–1521.
29. Perry, M.W., and Desplan, C. (2016). Love spots. Curr. Biol. *26*, R484–R485.

Current Biology, Volume *27*

Supplemental Information

A Novel Interception Strategy in a Miniature

Robber Fly with Extreme Visual Acuity

Trevor J. Wardill, Samuel T. Fabian, Ann C. Pettigrew, Doekele G. Stavenga, Karin Nordström, and Paloma T. Gonzalez-Bellido

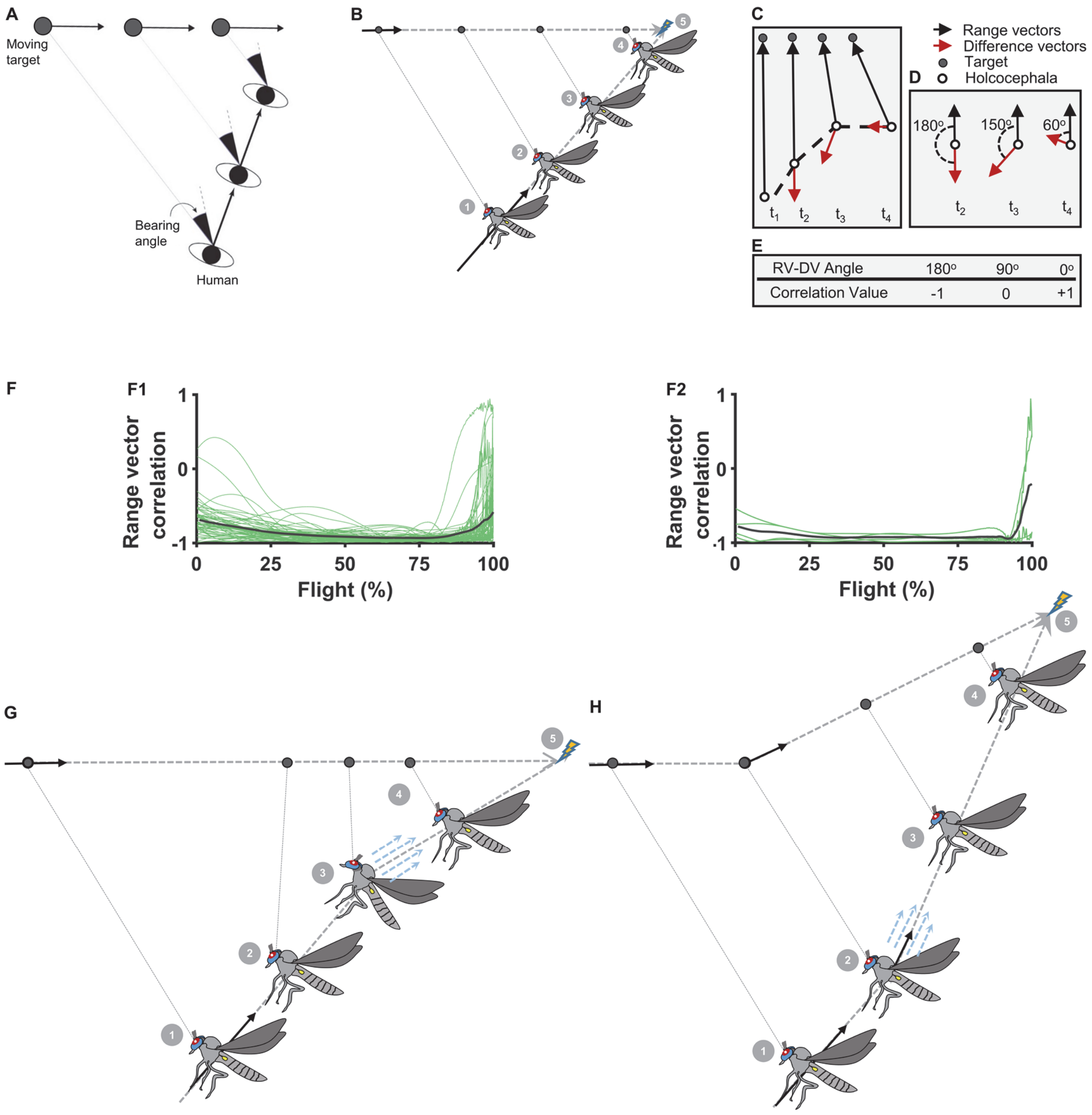

A
Moving target
Bearing angle
Human
B
C
Range vectors
Difference vectors
Target
Holcocephala
D
180º
150º
60º
E
RV-DV Angle
180º
90º
0º
Correlation Value
-1
0
+1
F
F1
F2
Range vector correlation
Flight (%)
G
H

Figure S1. Related to Figure 2. Diagrams showing how the constant bearing angle strategy (CBA) and proportional navigation can be used to intercept targets. (**A**) Top view diagram of human (head shown in black, shoulders in white) intercepting a ball moving on the ground. The constant bearing angle (CBA) strategy results in parallel range vectors (grey line between human and ball, also called the line of sight) that decrease in range. Reproduced from [S1], with permission from the author. (**B**) *Holcocephala* can also intercept a moving target by employing the constant bearing angle strategy: (1) When the prey flies with constant velocity and direction *Holcocephala* flies backwards to intercept it. (2) *Holcocephala* maintains its own heading and speed by maintaining the image of the prey on the same retina location. (3) This approach allows *Holcocephala* to maintain an interception heading, without explicitly knowing where the interception will take place. (4) At close range, the prey does not detect the *Holcocephala*, or does not have time to react. (5) The target is caught in a few more milliseconds. (**C**) To allow for comparison across studies, we also calculated the vector correlation as done by [S2]. For every frame in this trajectory, the difference vector is calculated (red). The difference vector is the difference between any one range vector and a previous range vector. (**D**) To calculate correlation between vectors, at every frame the range vector is subtracted form the difference vector (angle shown = RV - DV angle). As can be seen, when simply closing range and not changing angle, the difference vector is at 180° to the range vector. However, the difference vector becomes less than 180° when there are discrepancies in direction, as in t_3. When range is gained between fly and bead, the angle between the difference vector and the range vector falls below 90°. (**E**) The difference between the range vector (RV) and difference vector (DV) is then normalized (-1 to +1). This correlation value gives a proxy of how parallel the range vectors are between each other. (**F**) The resulting vector correlation value of trials in which the bead moved with a constant speed (F1) and those in which it decelerated or even reversed (F2). (**G**) Following alterations in the prey's bearing or velocity, proportional navigation can be used by *Holcocephala* to constantly update its own bearing and achieve interception without explicit knowledge of the time or point at which contact will occur: (1) The prey flies with constant velocity and direction. *Holcocephala* flies backwards to intercept it. (2) The target accelerates, and its image slips out of the acute zone. (3) *Holcocephala* compensates immediately by changing the tilt of its head, or the tilt of its head and body together. This action brings the image of the prey back to the acute zone. Simultaneously, the fly applies a proportional change to the rotation rate of its velocity vector, which results in a new heading direction. (4) *Holcocephala* stabilizes the range vector once more, and body position and the head are brought back to their original orientation. (5) The target is caught in a few more milliseconds. (**G**) The same process applies when a target changes direction: (1) The prey flies with constant velocity and direction. *Holcocephala* flies backwards to intercept it. (2) The prey changes bearing. (3) *Holcocephala* changes its own bearing proportionally. (4) The range vectors are maintained parallel throughout the trajectory. (5) The change in prey direction has been successfully nulled. The target is caught in a few more milliseconds.

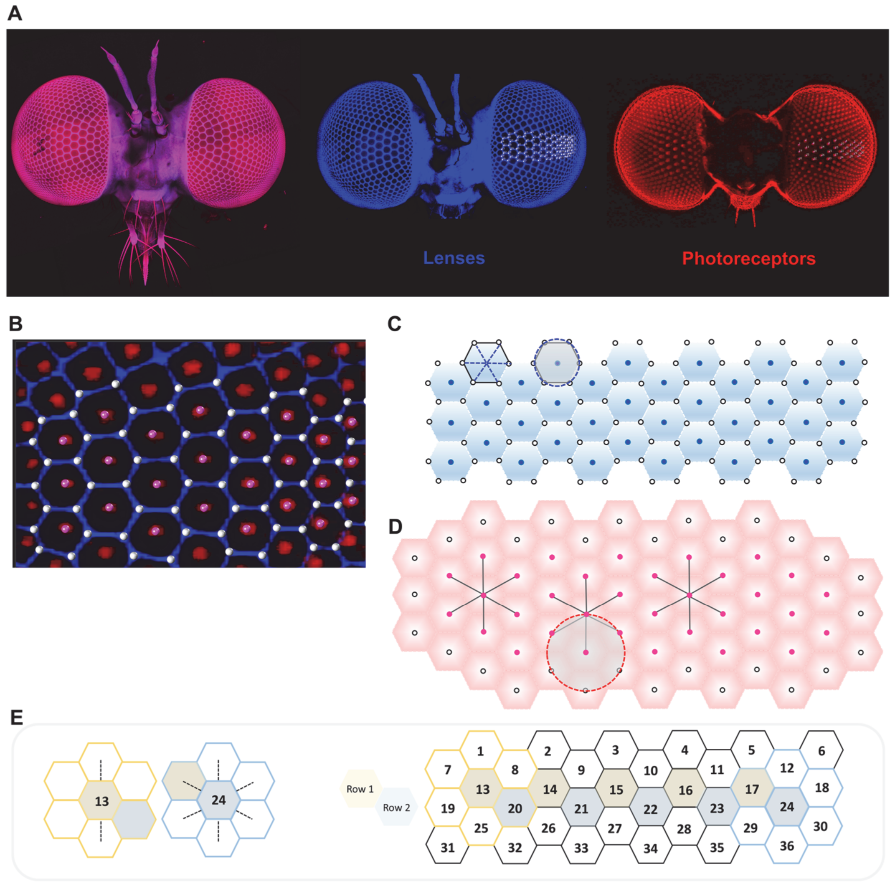

Figure S2. Related to Figure 4. Method for reconstructing interommatidial angles in *Holcocephala* through 2-photon microscopy. (**A**) Auto-fluorescence from a 3D stack of the *Holcocephala* head acquired with 2-photon microscopy. The markers placed on lenses and photoreceptors are displayed using data from a single colour channel with adjusted lookup tables to highlight features. (**B**) Close up image of a 3D stack with data from both red and blue channels displayed and overlaid with lens corner markers (white) and rhabdomere-tip markers (magenta). (**C**) The markers at the corners of each lens are used to calculate the lens diameter (average of 3 measurements, blue broken lines). Diagram showing how the centre of the lens (blue marker) is calculated as the centroid, or centre of mass, of the corner markers (i.e. average location of all 6 markers, blue dashed circle). (**D**) Diagram depicting how at the rhabdomere level, the ommatidia boundaries are not reliably detected. To reduce the error in the placement of the marker, the centroid method is employed: the centroid of the neighbouring rhabdomeres (red circle with dashed line) is used as the new rhabdomere location. In order to use this centroid method on all ommatidia, additional units need to be initially marked. The markers with recalculated locations are shown in magenta. This method yields “rosettes” formed by 1 centre ommatidium and 6 neighbouring ones (black lines), in which all the rhabdomere markers have been relocated. (**E**) Diagram showing how within each rosette, interommatidial angles are calculated as averages from either the vertical, or all neighbours. We placed sufficient markers to cover the entire fovea, and quantified 2 rows of 5 rosettes each.

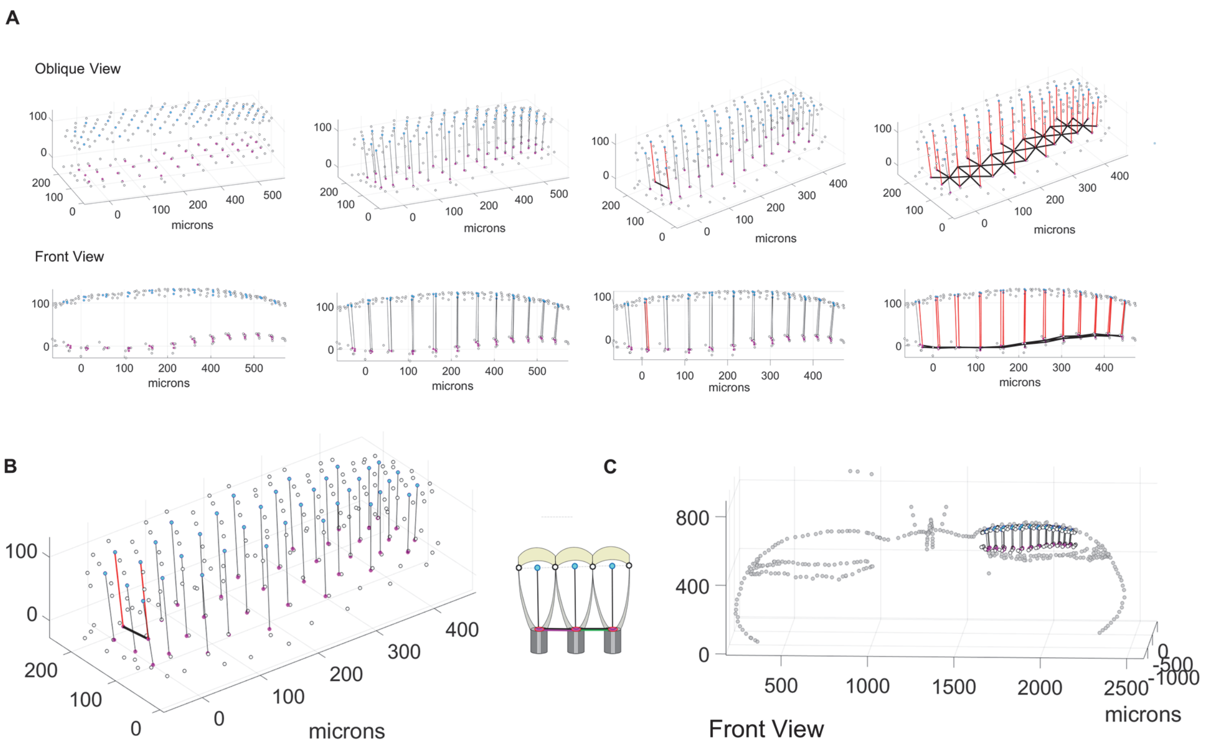

Figure S3. Related to Figure 4. Pipeline of the interommatidial angle reconstruction. (**A**) Figures showing the markers (as described in Extended Data Fig. S2B-E). In the cornea layer, white markers with a black edge are the lens corners and blue markers are the calculated lens centres. In the photoreceptor layer, white markers with a black edge are the original placement of the rhabdomere tips (as assessed by the experimenter) and pink markers are the recalculated location of the rhabdomere tips according to the centroid method. The visual axis of each ommatidium is given by the vertical line that joins the corresponding blue and pink markers. The interommatidial angle between any 2 units is calculated on the vertical plane that crosses the horizontal line (bold black) that joins them. This reduces the problem to 2D, and the angle between the two red vectors (the ommatidia axes) can be measured. (**B**) Magnified view showing that the relocation of the rhabdomere tips by the centroid method is minimal. (**C**) The reconstructed visual axes, within a digitized outline of the same *Holcocephala* head (grey markers).

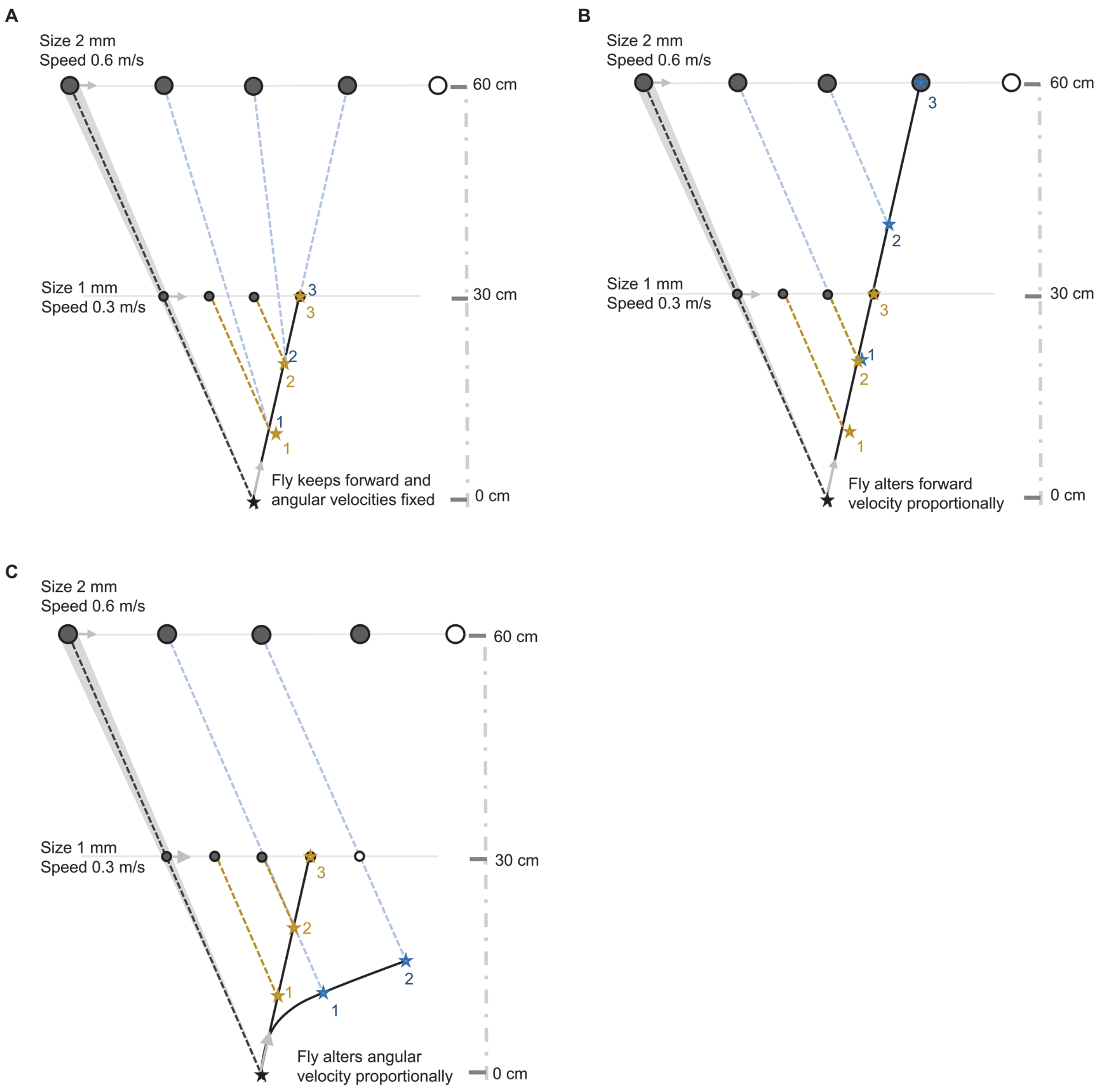

Figure S4. Related to Figure 2. Using the constant bearing angle strategy (CBA) during long range interception. (**A**) A 2 mm target is presented at 60 cm distance, but its size and distance are ambiguous to *Holcocephala* because this distance is outside of its depth perception range; it could be a slow moving small target that is close (1 mm bead) or it could be a fast moving large target that is far away (2 mm bead). *Holcocephala* starts with a speed appropriate to intercept the bead that is closer (1 mm bead). If this heading or velocity is not altered, the fly will “fall behind” and won’t be able to intercept the actual target (the 2 mm bead). (**B**) Once airborne, the fly can compensate for the size/distance discrepancy by applying a forward acceleration such that the average velocity doubles, yielding a constant bearing angle. This action will keep the range vectors parallel, and assure an interception. (**C**) As an alternative, the fly can change its heading, this will maintain the range vector parallel, and eventually ensure a trajectory that does not require a forward speed as high as that of (B), but the rate of closure will drop, which means that the interception will take much longer.

Supplemental Experimental Procedures

Animals

Collection, identification and filming were undertaken within the regional Parks in York, Pennsylvania. Permits for site access to perform the research and for sample collection were obtained from the local parks authority. Two *Holcocephala* species, *H. fusca* and *H. abdominalis*, share a common range within the USA and are functionally cryptic species [S3]. We refer to this species as *H. fusca*. Within this field site we also encountered *H. calva*, which were excluded from the experiments.

Computer controlled bead mover: Fly teaser

Beads were presented to *Holcocephala* using a custom made plastic frame that housed a stepper motor and several pulleys. The whole ensemble is referred to as the "fly teaser" (**Figure 1**). The beads were attached to a taut fishing line which was wrapped around the stepper motor pulley and moved past the pulleys on the apparatus. This allowed the precise movement of beads at designated velocities and timings. The stepper motor was computer controlled and the whole apparatus was on a monopod that acted as a handle and allowed us to hold the fly teaser near a chosen perched fly. To simplify the problems of 3D interactions, we aimed to align the approaching direction of the target with the body axis of the fly. For more information on the stepper motor controller see [S4]. For the experiments in which several consecutive beads of different diameters were used, we are confident that flies did not have to examine several targets at once, because the time between the end of head movements related to a rejected bead and the start of head movement to the following bead was 680 ± 63 ms (mean ± SE, n = 8), while the delay between the end of a head movement and take-off was in all cases < 350 ms.

Acquisition of behavioural data

To improve the contrast of the camera images and help us digitize the recorded trajectories, white fabric was placed 1-3 metres horizontally away from the fly and used in all experiments as a backdrop. Since the flies contrast their target against the sky, and given that the cameras were placed at right angles to the fly anterior-posterior body axis, it is unlikely that the background interfered with the prey capture behaviour of the animals. Data were acquired with two synchronized Photron SA2 cameras (Photron Limited, Tokyo, Japan) running at 1000 fps. The system was calibrated using the Matlab toolbox by J.Y. Bouguet's laboratory (Caltech, http://www.vision.caltech.edu/bouguetj/calib_doc/) with numerous alterations. The position of the prey (or bead) and that of the predatory fly was digitized with custom written Matlab scripts. The resulting XYZ positions were fed to a fitting algorithm for trajectory reconstruction [S5]. ** For a detailed method on how to calibrate, digitize and smooth such data, request the Analysis Package.*

Analysing the aerial attacks

For each trajectory, we noted the frame at which the flies started to take off as the first visible movement of the wings. We then calculated the distance from fly to bead at that frame. We had noted during experimentation which bead size was presented. From such data, we calculated the subtended speed and size.

LOS range vector correlation

From the recorded trajectories 63 were chosen for vector correlation because they represented complete pursuits of beads with stable trajectories that ended in a visible catch. Four trajectories were chosen for vector correlation as the target changed direction in mid-flight and the fly continued to pursue and visibly catch the bead. Range vectors between the bead and the fly were calculated, in two different ways. **Figure 2** shows the signed difference between any range vector and the median vector, whereas **Figure S1** shows the neighbouring vector correlation.

To calculate the "lock on" distance we searched for the location of the fly at the time that the fly began a terminal deceleration whilst approaching the target. To find the maximum distance at which flies would initiate a lock on phase (**Figure 2C2**), we used a sigmoidal function with 4 coefficients ($f(x) = a+(b-a)/(1+10^{((c-x)d)})$). To quickly gather suitable staring point values for each of the four coefficients, the sigm_fit matlab function (Ohad Gal at Matlab file exchange) was used. The minimum value possible (a) was fixed to zero. The maximum value (b), inflexion point (c) and slope (d) were estimated by best fit. The values obtained with Sig_fit for each coefficient (0, 288, 290, 0.0037) were used as starting points in the Matlab curve fitting GUI, with all four coefficients free to be estimated by best fit. In this plot n = 86 because trajectories with such geometry acquired later in the study, with beads of different sizes, are also included. To calculate the average "lock on" speed we took the mean fly speed in the last 10% of the flight as the speed had plateaued in the majority of flights by this point.

Acquisition of microscopy data

We took advantage of the miniature size of *H. fusca* and imaged two whole mounted heads (2.9 mm wide x 0.82 mm deep). Tissue processing was done as in [S6], but with an additional first step that involved bleaching the samples as described in [S7, 8]. Imaging was completed using an Olympus XLSLPLN25XGMP objective, a Newport Spectra-Physics InSight® DS+™ laser at 810 nm, and a Bruker (Prairie Technologies) In Vivo Microscope using GFP and RFP detection channels. The voxel resolution was 1.75 µm in X and Y and 1.8 µm in Z. To obtain the semi-thin head sagittal sections and ultrathin eye cross-sections (for TEM imaging) shown in **Figure 3B-C**, the specimens were prepared as outlined in [S9].

We first identified the ommatidia that laid at the eye midline, where the Semper cells or the photoreceptor caps were still present, and then imaged the first neighbouring ommatidium in which the rhabdomeres were visible [SSee 10 for anatomical scheme]. In this manner we guaranteed that the measurements were taken at the tip of the rhabdomeres, which is important because fly rhabdomeres diverge quickly below this level to minimize crosstalk [S11]. After fitting the outline of each rhabdomere with an ellipse, we calculated the distances from the R7 rhabdomere to the others rhabdomeres. Photoreceptor 3 is excluded from the comparison because the neural superposition wiring dictates that such rhabdomere is not an immediate neighbour [S12]. We then calculated the mean from the three ommatidia with the closest rhabdomeres.

To obtain the focal length measurements, we employed the cornea drop method [Sas described in 13]. Briefly, a cornea was cut and any leftover cells from the retina were gently removed with a brush. The cornea was then suspended in a drop of saline and the magnification of the focused image produced was used to calculate the focal length. To find the longest focal length in a robust fashion, we averaged the values from the longest 3 focal lengths within our sample.

Analysis of microscopy data
Stacks from the 2-photon microscope were stitched and converted with Fiji [S14] so that it could be loaded into Vaa3D [S15]. The lookup tables were altered to increase the signal from the lenses or the rhabdomeres. Vaa3D was used to mark the location of the corner of the lenses and the location of the tip of the rhabdomere group in the ommatidia of interest. Marker files were named as Fly27, representative of a small fly, and Fly29, representative of a large fly among those collected. They thus provide us with a range of measurements reflective of the biology. In each eye, we used the cornea and the photoreceptor cell signals (**Figure S2A**) to place markers (**Figure S2B-E**) for the 5 rows of ommatidia next to the head suture (used as a landmark) and which always contained the largest lenses. From these parameters we reconstructed the lens diameter and visual axis of each ommatidium (**Figure 4**, **Figure S3**). We did not mark and reconstruct the rest of the eye because (*i*) the measurement error would have been bigger since at present the software used does not allow cutting the 3D stack in an arbitrary plane, and this would be necessary for accurate placement of markers as the curvature of the eye increases, and (*ii*) it would create a high work load, but provide no answers about the finest acuity obtained in this eye. ** For a detailed method on how to mark the lenses and the rhabdomeres, please request the Analysis Package.*

Large errors in marker placement were nulled by the centroid method employed (**Figure S2**). With regards to deformation in the sample, the clearing agent used produces minimal shrinkage [Ssee 6]. Moreover, although we noticed that in some samples the lower half of the photoreceptors exhibited a bend, their tips were always held in place firmly, aided by the robust proximity to the cornea and pseudocone. To calculate the error associated with using this method to measure lens diameters, we looked into the size differences between the corresponding ommatidia in the left and the right eyes (we did this for the 2 heads). Such difference is a composite of the natural variation in lens size between the two eyes and the variability of our measurement, and therefore it provides a conservative estimate of measurement error. We found that the difference in diameter between corresponding ommatidia was 2.1 ± 1.6 µm for fly 27 and 1.0 ± 1.1 µm for fly 29 (mean ± SD, n = 36). Thus, the measurements for each of ommatidium was not significantly different between the two eyes (p-values from two tailed, paired student t-test: fly 27 and 29: 0.22 and 0.19, respectively; df = 35. On average, the measurement error was 2% and 4% of the total lens diameter.

Ideally, we would have compared our measurement of interommatidial angles with those obtained with the pseudopupil method. However, the *Holcocephala* pseudopupil cannot be seen due to the dark pigmentation of the eye. Therefore, to estimate the error associated with our method we compared the interommatidial angles obtained for the corresponding ommatidia in both eyes (as explained above for the lens diameter). Instead of simply giving an average for the rosette (one rosette is composed of a single ommatidium and all its immediate neighbours), we kept the vertical and diagonal interommatidial angles separate because a paired t-test for all the measurements showed that the diagonal interommatidial angles are larger than vertical ones (mean ± SE for $\Delta\varphi$ vertical: 0.62° ± 0.04, and $\Delta\varphi$ diagonal: 1.03 ± 0.09; p<0.001). The vertical interommatidial angles were not affected by the location of the ommatidia (i.e. medial, centre or lateral). Therefore, we treated all measured vertical interommatidial angles as one population, with mean difference between the two eyes 0.19° ± 0.03 (mean ± SE, n = 20 ommatidia). To calculate the final vertical interommatidial angle range for each unit, we averaged the corresponding value for both head samples and then applied ± 0.19°. Visual inspection confirmed that there was a natural cut off between the interommatidial angle of the

5 centre ommatidia and the rest (**Figure 4D,** red lined hexagons). Those 5 interommatidial angles have a mean range between 0.21 to 0.59°.

Stereopsis range

The stereopsis range for this animal from interommatidial angles and from the inter acute-zone distance was calculated by employing the equation developed by [S16].

$$E_{\infty} = b^2/\tan(\Delta\varphi/2)$$

where b is the inter acute-zone distance and $\Delta\varphi$ is the interommatidial angle. This equation yields the maximal distance that the robber fly can distinguish from infinity by binocular triangulation to be ~ 26 cm.

Analysis package available on request

The package contains the following analysis guides, the Matlab scripts necessary to analyse the data and a sample data set for each:

1. *How to mark lenses and rhabdomeres*
2. *How to calibrate, digitize, smooth data.*

Supplemental References

S1. Fajen, B.R. (2013). Guiding locomotion in complex, dynamic environments. Front. Behav. Neurosci. *7*, 85.

S2. Mischiati, M., Lin, H.T., Herold, P., Imler, E., Olberg, R., and Leonardo, A. (2015). Internal models direct dragonfly interception steering. Nature *517*, 333-338.

S3. Fisher, E. (2006). Communication posted on http://bugguide.net/node/view/67616#68560. (Calif. Dept. of Food & Agriculture).

S4. Wardill, T.J., Knowles, K., Barlow, L., Tapia, G., Nordstrom, K., Olberg, R.M., and Gonzalez-Bellido, P.T. (2015). The killer fly hunger games: Target size and speed predict decision to pursuit. Brain Behav. Evol. *86*, 28-37.

S5. Dey, B., and Krishnaprasad, P.S. (2012). Trajectory smoothing as a linear optimal control problem. 50th Annual Allerton Conference on Communication, Control, and Computing (Allerton), 1490-1497.

S6. Gonzalez-Bellido, P.T., and Wardill, T.J. (2012). Labeling and confocal imaging of neurons in thick invertebrate tissue samples. Cold Spring Harb Protoc *2012*, 969-983.

S7. Stockl, A.L., and Heinze, S. (2015). A clearer view of the insect brain-combining bleaching with standard whole-mount immunocytochemistry allows confocal imaging of pigment-covered brain areas for 3D reconstruction. Front. Neuroanat. *9*, 121.

S8. Smolla, M., Ruchty, M., Nagel, M., and Kleineidam, C.J. (2014). Clearing pigmented insect cuticle to investigate small insects' organs in situ using confocal laser-scanning microscopy (CLSM). Arthropod Struct. Dev. *43*, 175-181.

S9. Gonzalez-Bellido, P.T., Wardill, T.J., and Juusola, M. (2011). Compound eyes and retinal information processing in miniature dipteran species match their specific ecological demands. Proc. Natl. Acad. Sci. U.S.A. *108*, 4224-4229.

S10. Katz, B., and Minke, B. (2009). *Drosophila* photoreceptors and signaling mechanisms. Front. Cell Neurosci. *3*, 2.

S11. Wijngaard, W., and Stavenga, D.G. (1975). On optical crosstalk between fly rhabdomeres. Biol. Cybern. *18*, 61-67.

S12. Stavenga, D.G. (1975). The neural superposition eye and its optical demands. J. Comp. Physiol. *102*, 297-304.

S13. Stowasser, A., Rapaport, A., Layne, J.E., Morgan, R.C., and Buschbeck, E.K. (2010). Biological bifocal lenses with image separation. Curr. Biol. *20*, 1482-1486.

S14. Schindelin, J., Arganda-Carreras, I., Frise, E., Kaynig, V., Longair, M., Pietzsch, T., Preibisch, S., Rueden, C., Saalfeld, S., Schmid, B., et al. (2012). Fiji: an open-source platform for biological-image analysis. Nat. Methods *9*, 676-682.

S15. Peng, H., Bria, A., Zhou, Z., Iannello, G., and Long, F. (2014). Extensible visualization and analysis for multidimensional images using Vaa3D. Nat. Protoc. *9*, 193-208.

S16. Burkhardt, D., Darnhofer-Demar, B., and Fischer, K. (1973). Zum binokularen entfernungssehen der Insekten. 1. Die struktur des sehraums von synsekten. J. Comp. Physiol. A Neuroethol. Sens. Neural. Behav. Physiol. *87*.
